# Accumulation System: Distributed Neural Substrates of Perceptual Decision Making Revealed by fMRI Deconvolution

**DOI:** 10.1523/JNEUROSCI.1062-21.2022

**Published:** 2022-06-15

**Authors:** Yusuke Morito, Tsutomu Murata

**Affiliations:** ^1^Center for Information and Neural Networks, Advanced ICT Research Institute, National Institute of Information and Communications Technology (NICT), Suita, Osaka 565-0871, Japan; ^2^Center for Biosystems Dynamics Research, RIKEN, Kobe, Hyogo 650-0047, Japan

**Keywords:** category-selective region, decision making, deconvolution, evidence accumulation, fMRI, hierarchical cluster analysis

## Abstract

Neural substrates of evidence accumulation have been a central issue in decision-making studies because of the prominent success of the accumulation model in explaining a wide range of perceptual decision making. Since accumulation-shaped activities have been found in multiple brain regions, which are called accumulators, questions regarding functional relations among these accumulators are emerging. This study employed the deconvolution method of functional magnetic resonance imaging (fMRI) signals from human male and female participants during object-category decision tasks, taking advantage of the whole-brain coverage of fMRI with improved availability of temporal information of the deconvolved activity. We detected the accumulation activity in many non-category-selective regions (NCSRs) over the frontal, parietal, and temporal lobes as well as category-selective regions (CSRs) of the categorization task. Importantly, the frontal regions mostly showed activity peaks matching the decision timing (classified as “type-A accumulator”), while activity peaks of the parietal and temporal regions were behind the decision (classified as “type-B accumulator”). The CSRs showed activity peaks whose timing depended on both region and stimulus preference, plausibly reflecting the competition among the alternative choices (classified as “type-C accumulator”). The results suggest that these functionally heterogeneous accumulators form a system for evidence accumulation in which the type-A accumulator regions make decisions in a general manner while the type-B and type-C accumulator regions are employed depending on the modality and content of decision tasks. The concept of the accumulation system may provide a key to understanding the universality of the accumulation model over various kinds of decision tasks.

**SIGNIFICANCE STATEMENT** Perceptual decision making, such as deciding to walk or stop on seeing the signal colors, has been explained theoretically by the accumulation model, in which sensory information is accumulated to reach a certain threshold for making decisions. Neural substrates of this model, however, are still under elucidation among candidate regions found over the brain. We show here that, taking advantage of the whole-brain coverage of functional magnetic resonance imaging (fMRI) with improving availability of temporal information by deconvolution method, the accumulation is conducted by a system comprising many regions in different abstraction levels and only a part of these regions in the frontal cortex make decisions. The system concept may provide a key to explaining the universality of the accumulation model.

## Introduction

The behavioral properties of perceptual decision making have been successfully explained by the accumulation model, in which sensory information is temporally accumulated as evidence of each alternative, and one of the alternatives with evidence first reaching a certain threshold is selected (Ratcliff and Rouder, 1998; Ratcliff et al., 2016). (In this paper, we use the “accumulation model” to inclusively indicate the model variants.) Because of the remarkable effectiveness of the accumulation model in a wide range of decision making, neural substrates of the accumulation, which are referred to as accumulators and thought to show accumulation-like activity during decision processes, have been intensively studied (for recent review, see Brody and Hanks, 2016). Neurophysiological studies in monkeys have found accumulators in the lateral intraparietal area (Shadlen and Newsome, 2001), the frontal eye field (FEF; Kim and Shadlen, 1999), and the superior colliculus (Horwitz and Newsome, 1999). Functional magnetic resonance imaging (fMRI) studies have also found accumulators of the human brain, employing some approaches to overcome the low temporal resolution. In the accumulation-model-based approach as one of the typical approaches, accumulators were determined as regions whose blood oxygen level-dependent (BOLD) signals matched the predicted signals based on the modeled accumulation on even a finer timescale. The accumulators were found in multiple regions, such as the intraparietal sulcus including the human homolog of the lateral intraparietal area (Kayser et al., 2010; Liu and Pleskac, 2011), FEF (Liu and Pleskac, 2011), anterior insula (Grinband et al., 2006; Ho et al., 2009; Liu and Pleskac, 2011), medial frontal gyrus and subcortical structures (Grinband et al., 2006). In the temporal-profile approach as another one, the technique of gradually unmasking stimuli (James et al., 2000) was applied to make the decision process protracted enough for fMRI to track the associated signals. Innovatively by using a clustering method of temporal profiles of the BOLD signals, Ploran et al. (2007) classified the decision-related regions into three classes: accumulator, sensory processor, and moment-of-recognition region, the latter two of which were regarded as being associated with processes antecedent and subsequent to accumulation, respectively. In their results, many decision-related regions, which included most of the accumulators found by the accumulation-model-based approach described above, were classified as accumulator or moment-of-recognition region. Now that such multiple accumulators over the brain have been found, questions have arisen regarding their functional relations: How are these accumulators of different regions committed to decision making? It also remains to be clarified whether the moment-of-recognition regions of Ploran et al. (2007), which were mostly regarded as accumulators in the frontal cortex by the other studies, just follow decisions made by the other regions or are directly committed to making decisions. Another important issue has been raised by Tremel and Wheeler (2015) and Dunovan and Wheeler (2018), which found that the face-selective and house-selective visual regions in the category-decision task showed choice content-specific accumulation consistent with trial-based decisions. Thus, the relations between the content-specific accumulators and the other accumulators also have to be elucidated.

The present study addressed the issue of functional relations among the multiple accumulators in decision processes. Because the temporal evolution of neural activity plays an essential role in the accumulation account, we employed the fMRI deconvolution method, which recovered the approximate original neural activity from BOLD signals (Glover, 1999), aiming to improve the temporal-profile approach with keeping the fMRI advantage of the whole-brain coverage (Hanks and Summerfield, 2017). Using a visual category-decision task, we found that many regions over the frontal, parietal, and temporal lobes as well as the content-selective regions showed accumulation activities. However, only a certain class of these regions mostly in the frontal cortex showed activity peaks that matched decision moments, suggesting that the evidence accumulation is conducted by a system comprising multiple functionally-heterogeneous regions, only a part of which make decisions. We also discuss rationality of the system account to understand the generality of the accumulation model over various domains of decision making.

## Materials and Methods

### Participants

This study employed a cluster analysis of the decision-related activity as an essential procedure as described below. Thus, for the sample size of the study we do not have a readily usable method of power analysis suitable for the clustering which is exploratory rather than hypothesis based. Then we used a priori power analysis not for precise determination but as only guidance for the sample size. As our starting point, we followed the hypothesis by Ploran et al. (2007) that the class of sensory processor should be discriminated from the two classes of accumulator and moment-of-recognition region in terms of response-time dependence of activity peak times. In our results we found anatomically corresponding typical regions to these three classes, such as right middle occipital gyrus (R MOG) to sensory processor, right superior parietal lobule (R SPL) to accumulator, and left anterior insula (L AIns) to moment-of-recognition region. In our analysis in advance, for the first six participants whose response times (RTs) covered six 2-s bins from 1 to 13 s, (deconvolved), activity time courses of trials with each of the six response-time bins were averaged for the above three regions within each participant, and peak times of the averaged activity relative to the stimulus onsets were extracted. As response-time dependence indicators, SDs of the peak times across the six response-time bins were obtained for the three regions of the six participants. Effective sizes of Cohen's *d_z_* of the SD difference between paired regions were 1.10 and 0.90 for R SPL versus R MOG and L AIns versus R MOG, respectively, and a priori power analysis determined the respective minimum sample sizes of 7 and 10 with 80% power at a significance level of 0.05. Guided by these results, we determined the sample size to be >10. Fourteen healthy native Japanese speakers with normal or corrected-to-normal vision [nine women, aged 26.8 ± 7.8 (SD) years] participated in an fMRI experiment, which was composed of two sections: the first section investigated the decision process of categorizing degraded images (decision section) and the second section was to localize visual category-selective regions (CSRs) of individual participants (localization section). This study was approved by the Ethics Committee for Human and Animal Research of the National Institute of Information and Communications Technology, Japan. All participants provided written informed consent in accordance with the guidelines approved by this committee and were paid for their participation.

### Stimuli

**Figure 1.**
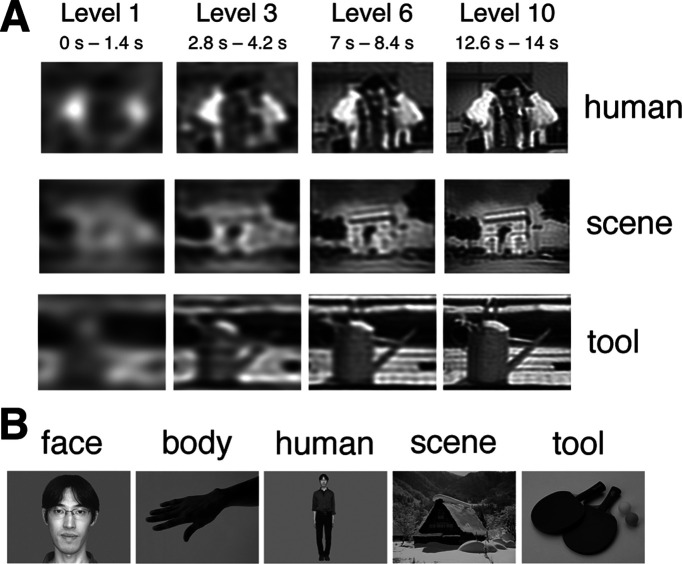
Examples of stimulus images. ***A***, Images used in the decision section, which were ones of three categories: human, scene, and tool. Although the original versions of the images were easy to categorize, each of them was low-pass filtered to produce 15-level degraded versions, which were sequentially presented by every 1.4 s from the most degraded level (level 1) to the least (level 15). The times below the levels indicate the presentation times from the stimulus onset. ***B***, Images used in the localization section, which were ones of five categories: face, body, human, scene, and tool. The backgrounds of the target objects were filled with uniform gray, except for the scene images. The mean luminance of each image was adjusted to be the same among all the stimulus images to avoid any effects caused by changes of stimulus luminance.

All the stimuli were digital photographic grayscale images of 640 (horizontal) × 480 pixels, whose original versions were selected from clip-art collections (Sozaijiten; Image Navi Corporation) and personal photographs. The images were presented using the software Presentation (Neurobehavioral Systems) onto a back-projection screen mounted outside the MRI scanner bore behind the participants' head, with subtending 8.0 × 6.0° of visual angle. In the decision section, we used 90 images consisting of 30 stimulus images of each of the three categories: human, scene, and tool. A “human” image depicted humans with both the head and body parts. A “scene” image included architectural scenery objects, such as houses and bridges. A “tool” image included handheld tools such as sports goods and stationery. We produced 15-level degraded versions of each image by means of spatial low-pass Fourier filtering, whose high cutoff frequency was manipulated from 1.0 to 4.5 cycles per degree (on the screen) with 15 levels of equal increments. Using the gradual unmasking technique (James et al., 2000; Carlson et al., 2006; Eger et al., 2007), the 15 versions were sequentially presented every 1.4 s from the most to the least degraded level (Fig. 1*A*). Participants were required to categorize the stimulus images as one of the three categories of human, scene, and tool. In the practice before the data collection, we also used 15-level degraded versions of another 20 images of each category.

In the localization section, we prepared 200 images consisting of 40 images of each of the five categories: face, body, human, scene, and tool. Each image included an exemplar object in the assigned category (Fig. 1*B*). A “face” image included a human face except other body parts, while a “body” image included parts or a whole of a human body except a head. A “human” image included a whole human body with head. A “scene” image includes natural scenery objects such as mountains or the same types of architectural scenery objects as in the decision section. A “tool” image included the same types of handheld tools as in the decision section. The backgrounds of the target objects were filled with uniform gray, except for the scene images, whose backgrounds were kept as the important content of the expanse of scenery. Unlike the decision section, all the images were not degraded but were clear enough to recognize the depicted objects easily and unambiguously.

### Acquisition and preprocessing of MRI data

MRI images were acquired using a 3T MR scanner (Trio, Siemens). For functional imaging, an ascending T2*-weighted gradient-echo echoplanar imaging (EPI) procedure was used to produce trans-axial slices covering the entire cerebrum and cerebellum, excluding the eyeballs, by oblique scanning. Imaging parameters in the decision section were repetition time (TR) = 2000 ms, echo time (TE) = 30 ms, flip angle (FA) = 79°, field of view (FOV) = 192 mm, 64 × 64 matrix, 34 slices, and voxel size = 3.0 × 3.0 × 3.45 mm. In the localization section, TR = 2500 ms, TE = 30 ms, FA = 80°, FOV = 192 mm, 64 × 64 matrix, 38 slices, and voxel size = 3.0 × 3.0 × 3.0 mm. To ensure stabilization of the functional imaging data, the first five scans (10 s) of the decision section and the first four scans (10 s) of the localization section were discarded (not used in the subsequent analyses).

The acquired MRI data of raw DICOM format were converted to NIFTI format and preprocessed using the software Statistical Parametric Mapping 12 (SPM12; Friston et al., 1995; Wellcome Trust Center for Neuroimaging, London, United Kingdom; https://www.fil.ion.ucl.ac.uk/spm), implemented in MATLAB 2016b (MathWorks). For the data of each session, the displacement of scans caused by the participants' head motion was realigned relative to the mean of the images using the affine transformation, modifying the headers of the images to reflect their relative displacement. In the determination of regions of interest (ROIs), to improve the accuracy of blocked-design SPM analysis, we applied the slice timing correction, which concerns differences in signal acquisition times because of the positions of slices and modifies each time series in the slice to have values that would have been obtained if the slice had been acquired at the same time as the reference slice. The reference slices were the middles of the scans, which were 17 and 19 in the decision and localization sections, respectively. In the temporal-profile analysis of data of the decision section, however, we did not apply the slice timing correction; instead, we conducted the slice-timing modeling by which we obtained not-modified values of fMRI data with their accurate acquisition times (Kiebel et al., 2007) as described below in the subsection, Profile classification of deconvolved signals. Spatial normalization was applied to the data with nonlinear three-dimensional transformations into the ICBM template of East Asian brains, which we used because all the participants were native Asian (Japanese). The normalized images were spatially smoothed in a three-dimensional Gaussian kernel with a half-maximum width of 6 mm.

### Experimental sections

**Figure 2.**
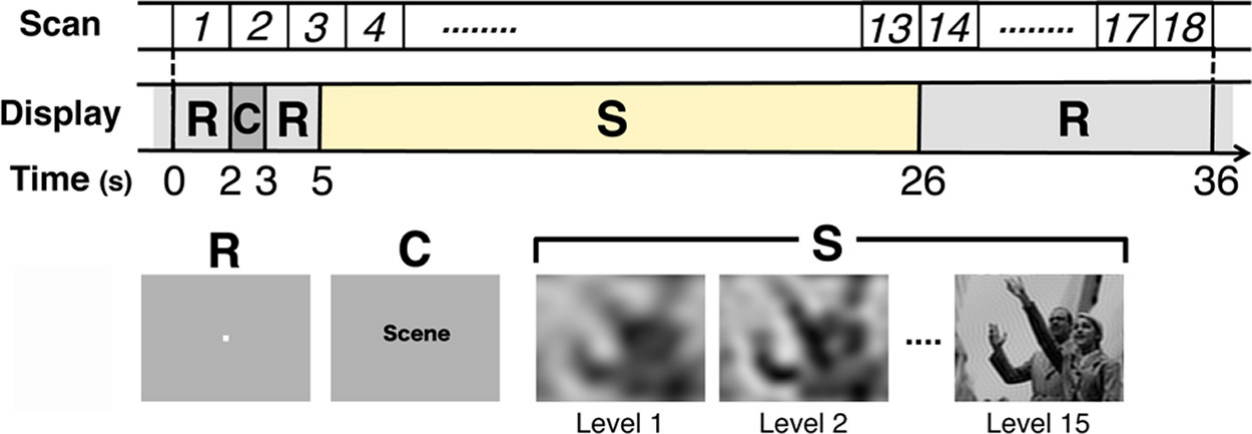
Design of a trial of the decision section. Each trial lasted for 36 s, which was synchronized with the 18 scans of fMRI with a TR of 2 s. R and C indicate the epochs of rest and cue, respectively. In the stimulus epoch indicated by S, whose onset (at 5 s) was at the midpoint of the third scan, a set of 15-level degraded versions of a stimulus image were sequentially presented with a changing interval of 1.4 s causing little synchrony with the scan timing.

Each participant had the decision section first and the localization section on a different day to prevent fatigue. The decision section consisted of 90 trials, which were given in six sessions of 15 trials each. One stimulus image was used in each trial, and 90 trials covered 30 images for each of the three categories (human, scene, and tool). As shown in Figure 2, each trial lasted for 36 s, which began with a 2-s rest epoch in which a white fixation point in the center of a uniform gray background was presented, followed by a 1-s cue epoch in which a black word of the cue for the oncoming stimulus was presented. Intervened by a 2-s rest epoch, a stimulus epoch lasted from 5 s to 26 s, in which a set of 15-level degraded versions of a stimulus image were sequentially presented every 1.4 s according to the gradual unmasking technique. The stimulus epoch was followed by a 12-s rest epoch, which was composed of the remaining 10 s of the present trial and 2 s at the beginning of the next trial. This length of the rest epoch was expected to be long enough for the BOLD signal elevated in the preceding stimulus epoch to approach the baseline levels (Tremel and Wheeler, 2015). The beginning of a trial was precisely synchronized with the fMRI scan with a TR of 2 s, so that a trial comprised 18 scans and the stimulus onset (at 5 s) was at the midpoint of the third scan of the trial. The least common multiple of the TR (2 s) and the unmasking step interval (1.4 s) was large enough (14 s) so that the image-change moments of the unmasking steps were little synchronized with the scan timing. A cue word was given ahead of each stimulus epoch to investigate the influence of prior knowledge on categorical decisions. We expected that a cue congruent with its following stimulus would facilitate decision while an incongruent cue would obstruct the decision to the contrary, so that cue congruency was expected to influence the accumulation of the decision process. Either “Human,” “Scene,” “Tool,” or “****” (indicating no cue) was displayed in characters at the center of the screen with a size around 5° of visual angle. (Quotation marks, “ ”, are not displayed.) To evaluate the effects of cues, we gave stimulus-congruent cues, incongruent cues, and no cues, each of which were in one third of all the trials; for example, 30 epochs of human stimuli were preceded by “Human” in 10 trials, “Scene” and “Tool” in five trials each, and “****” in 10 trials. The presentation order of the cue-stimulus combinations was randomized. Participants were required to discriminate the stimulus category among human, scene, and tool and respond by clicking the assigned button as soon as they could discriminate it. The buttons for the index, middle, and ring fingers were assigned to human, scene, and tool, respectively. We encouraged participants not to hesitate to respond by allowing the second clicks to revise their answers. Participants were told that only clicks during a stimulus epoch were valid. As a result, 68% of all trials across participants responded correctly in the first response, and in 96% of all the trials correct answers were obtained in the final (including the second and later) responses, showing that most of the stimuli with the least degradation could be correctly categorized. In the following fMRI time series analysis, we used only the trials with the first correct responses not to intermingle the activity of the second-decision process. To monitor the subjective verification of the participants' recognition, the instruction asked participants to double-click the same button as their previous final response at any time within the stimulus epoch when they became confident of the answer. The results of the present study showed that participants verified their responses in 86% of all the correct-first-response trials, indicating that participants consistently recognized the images through the stimulus epochs of most of the correct trials. Relative temporal positions of the verification-RTs were shown to be almost uniformly distributed in the intervals between the correct first response and the end of stimulus epoch of trials with a mean of 0.54 and SD of 0.25 (across trials and participants) by comparing with a theoretical uniform distribution that has the mean of 0.50 and SD of 0.29. Thus, influences of the verification responses on activity profiles seem to be minimal. One session comprised 278 scans (556 s), the initial five scans of which were discarded. The remaining 273 scans, which consisted of 270 scans of 15 trials by 18 scans and an additional three scans after the last trial, were used in the following analysis. A decision section typically took ∼100 min for the data collection, including breaks inside the MRI scanner and ∼50 min for instructions, practice using another 20 images of each category, and postexperiment debriefing outside the scanner.

The localization section consisted of four sessions, each of which included 10 trials. Each trial was dedicated to one of the five categories of face, body, human, scene, and tool, and eight trials for each category were conducted in a randomized order. Each trial began with a 2-s rest epoch (only a fixation point), followed by an 18-s stimulus epoch and a subsequent 10-s rest epoch, constituting a 30-s duration. During a stimulus epoch, the first image was presented for 2 s followed by a 1-s fixation point, and 10 images of the same category were presented in successive turns every 1.5 s. The first image appeared once or twice again in the subsequent 10 images of randomly selected turns. All the other nine or eight images were different. To enhance attention to the stimuli, participants were required to remember the first image and to press an assigned button when the same image appeared again within the stimulus epoch. Each of the 200 images was used twice in this section, covering the 400 images used in the 40 trials in total. Each trial began in synchrony with the fMRI scan, with a TR of 2.5 s. One session comprised 126 scans (315 s), the initial four scans of which were discarded. The remaining 122 scans, which consisted of 120 scans of 10 trials by 12 scans (30 s) and an additional two scans after the last trial, were used in the following analysis. A localization section for a participant typically took ∼70 min including instructions, practice, short breaks, and debriefing.

### Determination of ROIs

**Figure 3.**
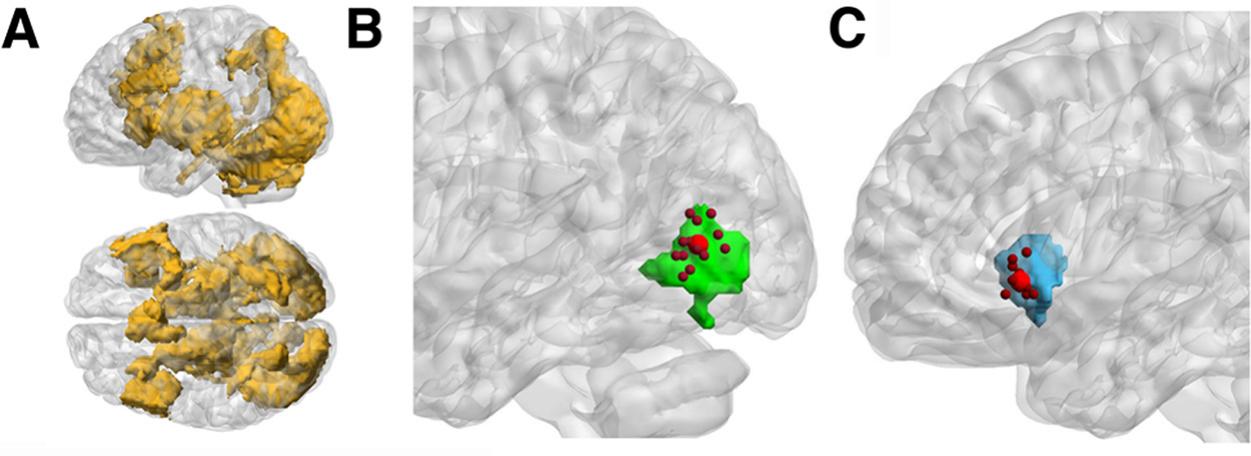
Procedure to determine coordinates of ROIs. ***A***, The mask of positive activation, which consists of all the voxels detected in the group-level analysis of positive activation throughout stimulus epochs of the decision section (cluster-wise threshold of the FWER *p* < 0.05). ***B***, An example of coordinates of a CSR: left body-selective region. The light green region indicates a body-selective region, which was obtained in a group-level analysis (FWER *p* < 0.05) with inclusive application of the mask of positive activation. Following the “individual peaks within group activation” method (O'Reilly et al., 2012), within this region, the peak voxels of body-selective activation of all the participants were extracted (small dark red spheres) from the individual-level *t*-contrast images (voxel-wise threshold *p* < 0.001, uncorrected). Their mean coordinates (large red sphere) represent the location of this body-selective region. The SD of coordinates of participants was 7.4 mm in this example of our data. ***C***, An example of coordinates of an NCSR: the left anterior insula. The light blue region indicates the left anterior insula of the TPM atlas masked inclusively by both the masks of positive activation and noncategory selectivity. Within this region, the peak voxels of decision task-related activation of participants were extracted (small dark red spheres) from the individual-level *t*-contrast images (voxel-wise threshold *p* < 0.001, uncorrected) obtained from the decision-section data. Their mean coordinates (large red sphere) represent the location of the left anterior insula. The SD of coordinates of participants was 4.8 mm in this example of our data.

As for regions associated with perceptual decision making, we determined ROIs that showed the decision task-related positive activation. To determine coordinates of each ROI, we adopted “individual peaks within group activation” method (O'Reilly et al., 2012), in which we selected the voxel that showed the strongest task effect in the individual-level analysis of each participant with applying an inclusive mask that was the activation ROI detected by the group-level analysis. This approach provides the most sensitive results of the individual-level effect, allowing for interindividual differences within the functionally identical ROI. Using the preprocessed (slice-timing corrected) fMRI data, the following three steps were conducted using SPM12. In the first step, using the decision section data, we determined positively activated regions during the decision process regardless of the stimulus category, to use it as an inclusive mask of positive activation. The reason to restrict ROIs to positive activation was that the accumulator regions, which were the primary targets of this study, should show positively-growing activity during the decision process. We defined a model of task-related positive activation as being positive relative to the baseline throughout all the stimulus epochs of the decision section; thus, the model of positive activation was common to all the sessions, whose stimulus epochs had the same temporal structures. This model was convolved with the canonical hemodynamic response function (HRF; Friston et al., 1998) for use as the regressor of positive activation in a blocked design SPM analysis. In the individual-level (first-level) analysis, the fMRI data of each participant were analyzed by a general linear model (GLM) using the regressors, applying high-pass filtering with a cutoff period of 128 s, and serial autocorrelation estimation with a first-order autoregressive model to eliminate artifactual low-frequency trends and aliased biorhythms. A single-condition contrast of the positive activation was applied to the parameter weights (β values) of the regressor, and a *t*-contrast image of an individual participant was constructed. The *t*-contrast images of all participants were incorporated into a random-effects model to make inferences of a group-level (second-level) analysis (Holmes and Friston, 1998). Activation was detected with a voxel-wise threshold of *p* < 0.001 (uncorrected for multiple comparisons) and a cluster-wise threshold of the familywise error rate (FWER) *p* < 0.05, corrected for multiple comparisons based on random field theory (Friston et al., 1994b), which corresponded to a cluster-size threshold of 1257. Then, a spatial mask of positive activation was made of all the voxels detected with this cluster-wise threshold of the group-level analysis (Fig. 3*A*), and used as an inclusive mask common to all the individual participants in the following steps.

In the second and third steps, choice-selective and nonchoice-selective regions were determined. The choice-selective regions of this study were visual CSRs because the task of the decision section was to discriminate the object categories. The nonchoice-selective regions, to which we refer as non-CSRs (NCSRs), were defined as all regions that did not show any selectivity for the concerned categories. The second step was to determine the coordinates of the CSRs of individual participants using data from the localization section. In the SPM analysis, we modeled five regressors at the individual level representing the stimulus epochs of the five categories: face (F), body (B), human (H), scene (S), and tool (T). The GLM of these regressors was applied to the preprocessed data of the localization section, including the high-pass filtering and serial autocorrelation estimation in the same way as in the first step. The *t*-contrast images of category selectivity were obtained using four contrasts of the weighted regressors, which were designed according to the previous studies of CSRs as follows: F > (S + T)/2 for face selectivity (Kanwisher et al., 1997), B > (S + T)/2 for body selectivity (Downing et al., 2001), S > (H + T)/2 for scene selectivity (Epstein and Kanwisher, 1998), and T > (H + S)/2 for tool selectivity (Grill-Spector et al., 1999; Chao et al., 1999). Here, we used the regressor of H to define scene and tool selectivity because it is not clear whether the F-regressors and B-regressors, objects of whose categories are evidently related, can be used equivalently with the T-regressor or S-regressor in making the contrasts. With the H-regressor, all the kinds of selectivity could be defined by contrasting with two other categories in a consistent manner as above. For each category selectivity, the individual-level *t*-contrast images of all participants were incorporated into a group-level analysis with inclusive application of the positive-activation mask. The selective regions were detected with a voxel-wise threshold of *p* < 0.001 (uncorrected) and a cluster-wise threshold of the FWER (*p* < 0.05, corrected for multiple comparisons), which corresponded to cluster-size thresholds of 90, 80, 161, and 921 for selectivity of face, body, scene, and tool, respectively. Since separate (discontiguous) clusters of voxels detected at the group level with even the same contrast could be distinct functional regions of that category selectivity, each contiguous cluster was regarded as a distinct CSR, resulting in 3, 5, 5, and 1 regions selective for face, body, scene, and tool, respectively. Each of these CSRs was applied as an inclusive mask to the individual-level *t*-contrast image of each participant regarding the corresponding category selectivity, and we extracted all the local peak voxels whose *t*-values were above the voxel-wise threshold *p* < 0.001 (uncorrected). In six of the 14 CSRs above, two or more of the 14 participants showed no significant voxels; thus, we excluded these six CSRs from the following analysis. For the remaining eight CSRs, which were two face-selective, two body-selective, three scene-selective, and one tool-selective region, the Montreal Neurologic Institute (MNI) coordinates of each region were determined for each participant by selecting the peak voxel that showed the maximum *t*-value for its selectivity condition. Since in the later stages we averaged activity signals from the voxels of the participants in the same region, we expected that reducing the dispersion of the locations among participants of an identical region would contribute to lowering functional variability and clarifying common activity profiles among participants. Thus, to diminish the spatial dispersion of the coordinates among participants, we conducted an additional operation in which the location of a participant with the largest deviation from the mean location of all the participants was replaced with the peak of the next greatest *t*-value of that participant (only when the participant had two or more peaks) if this replacement reduced the variability of the locations. The replacement was allowed to repeat for a participant who showed the largest deviation among participants until no reduction in the variability was obtained. As a result, 11 peaks (10%) of 112 regions (8 CSRs by 14 participants) were replaced by the second greatest peaks, and this operation reduced the SD of the coordinates among participants by 0.7 mm, resulting in 7.8 mm on average of the eight CSRs. The location of each region was represented by the mean coordinates of all the participants of that region (Fig. 3*B*).

In the third step, by inversely using the category-selective maps, we defined NCSRs that showed positive activation without any significant category selectivity. The second step produced 56 images of category-selective activation in the individual-level analyses for four categories by 14 participants with a voxel-wise threshold of *p* < 0.001 (uncorrected). Using the Image Calculator of SPM12, these 56 images were summed up and inverted (by taking 1 for 0 and 0 for non-zero values of the summed image) to obtain a binary image, all of whose voxels did not show any category-selective activation of any participant with such a liberal threshold. This image was used as a spatial mask for noncategory selectivity. To parcel the noncategory-selective range, which extends over the whole brain, into the anatomic structures, we employed the anatomic atlas of the tissue probability map (TPM) available with SPM12. The TPM provides the MNI-space volume data of 136 tissue structure-based regions of the brain, including both neural and non-neural types. Excluding 15 structures that lack neuronal cell bodies, such as white matter, optic chiasm, vessels, and ventricles, we used the volume data of 121 atlas regions of cortical areas and nuclei. The intersection of each of these TPM-atlas regions and both masks of noncategory selectivity and positive activation provided a TPM-based NCSR that showed positive activation during the decision task. We refer to this volume of intersection simply as an NCSR. Each of these 121 NCSRs was applied as an inclusive mask to the individual-level *t*-contrast images of the task-related positive activation that were obtained from the decision-section data in the first step, and we extracted all the local peak voxels whose *t*-values were above the voxel-wise threshold *p* < 0.001 (uncorrected). In 66 of the 121 NCSRs, two or more of the 14 participants showed no significant voxels; thus, we excluded these 66 regions from the following analysis. We determined the MNI coordinates of each of the remaining 55 NCSRs for each participant in the same way as the CSRs; that is, the coordinates of the voxel with the maximum *t*-value were adopted, followed by an additional operation in which the voxel of a participant deviated most from the mean location of all participants was replaced with a voxel of the second greatest *t*-value of that participant if this replacement reduced the location variability. As a result, 137 peaks (18%) of 770 regions (55 NCSRs by 14 participants) were replaced by the second greatest peaks, and 10 peaks (1%) were replaced by the third or fourth greatest peaks. This operation improved cohesiveness of the peak locations among participants, reducing the coordinates SD by 2.5 mm resulting in 5.0 mm on average of the 55 NCSRs. The location of each region was represented by the mean coordinates of all the participants of that region (Fig. 3*C*).

### Deconvolution of BOLD signals

Using the preprocessed, normalized, but not slice-timing corrected fMRI data of the decision section, BOLD signals were extracted from voxels at the coordinates of the eight CSRs and 55 NCSRs determined above for each participant. The BOLD time series of each session (273 data points) was linearly detrended, keeping its mean value, and we estimated its baseline level with the mean of 45 signal values of the first, second, and 18th scans of the 15 trials, which were temporally the most remote rest scans from the previous stimulus onsets. We used the 271st and 272nd scans just after the last (15th) trial instead of the first two scans of the first trial, expecting the baseline activity to be more stabilized. Using this baseline, the time series was scaled to percent signal change (PSC) from the baseline. Here, we introduce deconvolution of the PSC of BOLD signals aiming to improve availability of temporal information of the signals on the following grounds. Because of the linearity of the hemodynamic system, a BOLD signal can be well approximated by convolving the neural activity with a HRF that is an impulse response (Friston et al., 1994a; Boynton et al., 1996). Since the convolution makes the BOLD signal delayed and blurred from the original neural activity, it is difficult to determine the timing of the original activity using the BOLD signal in a straight manner. On the other hand, the accumulators are characterized by their temporal profiles of activity regarding the onsets and peaks relative to the stimulus onsets and subject's responses. Therefore, recovery of the neural activity from BOLD signals can be expected to be effective in searching for the accumulators, and we employed Wiener deconvolution as one of the powerful methods for such recovery. The PSC time series was used to estimate the original neural activity using the Wiener deconvolution (Papoulis, 1977; Glover, 1999; David et al., 2008; Surhone et al., 2011; Wu et al., 2013) with the following details.

Let s(t) and h(t) be the time series of neural activity and HRF, respectively. The measured time series of the BOLD signal in PSC, m(t), originating from the neural activity, is expressed as following:

m(t)=h(t)∗s(t) + n(t),

where ∗ denotes the convolution operation of the neural activity and HRF, and n(t) denotes the noise in the measurement. Using the Wiener deconvolution filter w(t), the neural activity is estimated as $\hat{s}(t)$ from the BOLD signal m(t) as follows:

$$\hat{s}=w(t)*m(t).$$

According to the Wiener deconvolution theory,

$$W(f)=\frac{1}{H(f)}\left[\frac{{\left|H(f)\right|}^{2}{\left|S(f)\right|}^{2}}{{\left|H(f)\right|}^{2}{\left|S(f)\right|}^{2}+{\left|N(f)\right|}^{2}}\right],$$

and

(1) $$\hat{s}(t)=IFT\left[W(f)M(f)\right],$$

where W(f), M(f), H(f), S(f), and N(f) are the Fourier transforms of w(t), m(t), h(t), s(t), and n(t), respectively, and the squared forms such as ${\left|N(f)\right|}^{2}$ are the energy spectral density (ESD) of the corresponding quantity. IFT indicates the inverse Fourier transformation. For simplicity, we also refer to W(f), the Fourier transform of the Wiener filter w(t), as the Wiener filter. Postulating that the neural activity (signal) and noise are uncorrelated, then

$${\left|M(f)\right|}^{2}={\left|H(f)\right|}^{2}{\left|S(f)\right|}^{2}+{\left|N(f)\right|}^{2},$$

and using this relation we obtain

(2) $$W(f)=\frac{1}{H(f)}\left[1-\frac{{\left|N(f)\right|}^{2}}{{\left|M(f)\right|}^{2}}\right].$$

This indicates that the Wiener filter attenuates the Fourier components with larger ratios of the noise relative to the measured signal, consequently diminishing noise in the deconvolved signal. Thus, using the appropriate functions of H(f) and N(f), we can obtain an approximation of s(t) from the measured data m(t).

**Figure 4.**
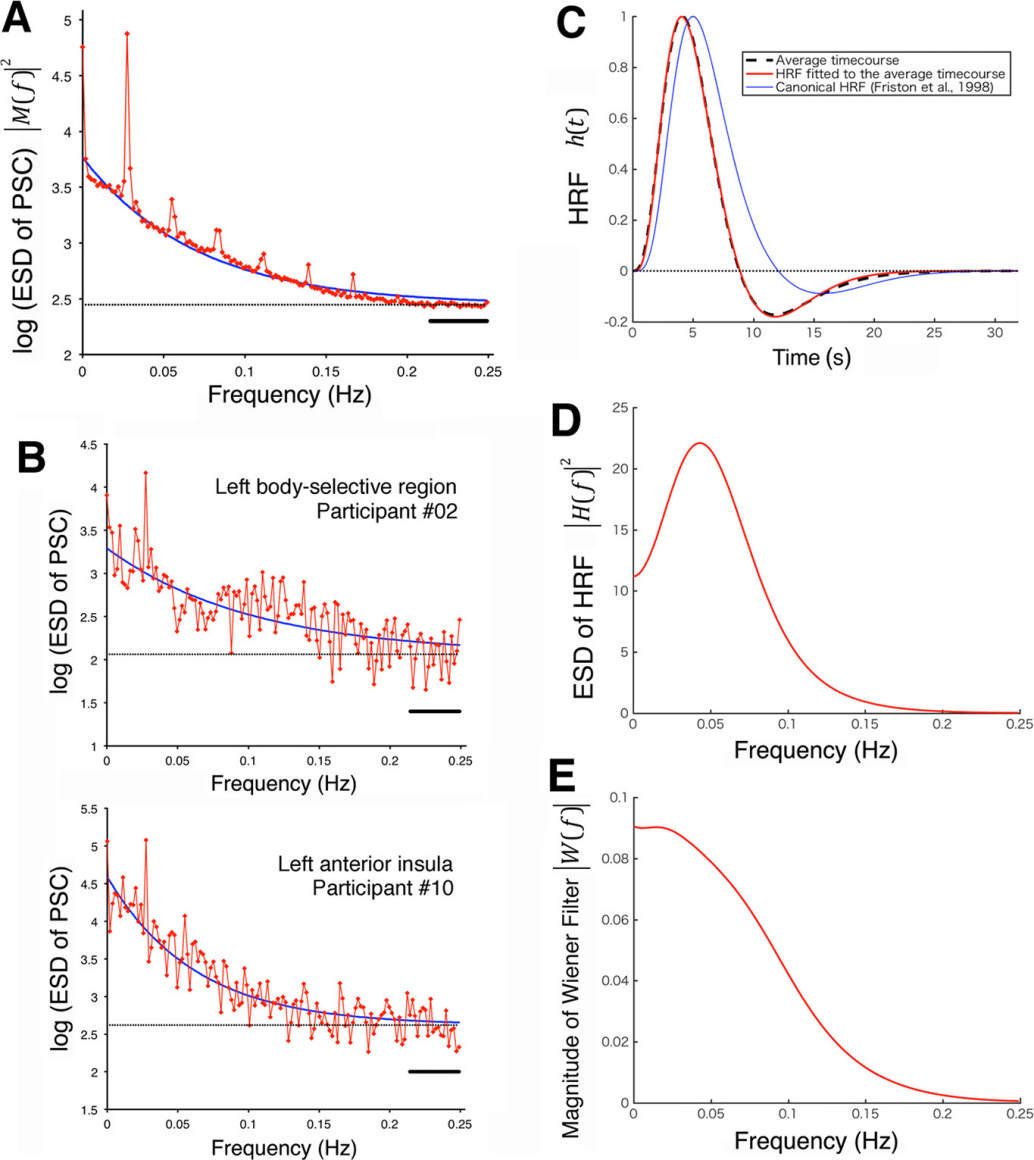
Construction of the Wiener filter. ***A***, The red symbols with lines indicate the one-sided ESD ${\left|M(f)\right|}^{2}$ of the signals in PSC on a logarithmic scale, grand-averaged across all the sessions over regions and participants. The frequency ranged from 0 Hz (DC component) to 0.2491 Hz with the interval of 1/546 Hz, given by the Fourier transformation of the time series of 273 sample points with TR of 2 s. The values at the 20 highest frequencies (from 0.2143 to 0.2491 Hz), whose range is indicated by the black thick line, were averaged to estimate the noise energy $\log N_{0}^{2}$, whose magnitude is indicated by the black dotted line. The period of a trial (36 s) caused the outstanding increases at the harmonics of 1/36 Hz. Excluding these harmonics of the trial frequency (3 points centering at each peak of the first to sixth harmonics) and the DC component (at 0 Hz), we fitted $\log{\left|M(f)\right|}^{2}-\log N_{0}^{2}$ with an exponential function Cexp(−Df) by means of the least square's method. The result showed a successful fitting (*r*^2^ = 0.986) with *C* = 1.32 and *D* = 14.3 s. ***B***, Examples of the ESDs of individual regions of individual participants. The noise energy estimation and fitting of the exponential functions were conducted in the same way as ***A***. First example: the left body-selective region of participant #02. *C* = 1.23, *D* = 9.8 s, *r*^2^ = 0.63. Second: the left anterior insula of participant #10. *C* = 1.97, *D* = 16.1 s, *r*^2^ = 0.86. ***C***, Broken line, The average time course of the model functions fitted to the 62 samples of the Handwerker dataset of HRFs. Red line, The model function fitted to the average time course, which was used as the dataset-based empirical HRF in the Wiener deconvolution (see the main text for the parameters). Blue line, For reference, the canonical HRF (Friston et al., 1998; provided by SPM12) with the default shape parameters ($\alpha_{1}$= 6, $\alpha_{2}$ = 16) and normalized amplitude (*A*_1_ = 5.70, *A*_2_ = −0.95). The dotted line indicates the zero-baseline. All the functions were calculated with the time step of 0.1 s and normalized to start at 0 and peak at 1. ***D***, The two-sided ESD of the empirical HRF of (***C***) ${\left|H(f)\right|}^{2}$, which was used in the Wiener filter. (The function is shown on the lower half of the frequency axis.) ***E***, The magnitude of the Wiener filter |W(f)| of Equation 3, where δ = 24. This panel shows the Wiener filter obtained by using the function $N_{0}^{2}/{\left|M(f)\right|}^{2}$ of the grand-averaged ESD (panel ***A***), whereas in the deconvolution of data the filter was obtained for each region of each participant.

The measured data M(f) at high frequencies seem to be dominated by the noise component N(f) because the HRF works as a temporal low-pass filter that substantially cuts off high-frequency components of neural activity. As shown in Figure 4*A*, this property was verified by the ESD ${\left|M(f)\right|}^{2}$ of the PSC time series of this study, averaged across all the sessions over regions and participants, which displays the flat energy content over high frequencies above ∼0.2 Hz suggestive of the constant noise components. This grand-averaged ESD (Fig. 4*A*) was used to estimate the mathematical form of ${\left|M(f)\right|}^{2}$, which was applied to data of individual regions below. Following Glover (1999), we assumed a constant spectrum of noise (white noise) as ${\left|N(f)\right|}^{2}=N_{0}^{2}$, whose magnitude was estimated from the high-frequency components of ${\left|M(f)\right|}^{2}$. We estimated the noise energy log*N*_0_^2^ by averaging the values at the 20 highest frequencies above 0.214 Hz, as indicated by the thick line in Figure 4*A*. As a typical function describing this type of data distribution, we fitted an exponential function with the asymptote $\log N_{0}^{2}$:

log|M(f)|2=Cexp(−Df)+log N02,

to the ESD data by means of the least squares method, excluding the DC component (*f* = 0 Hz) and the harmonics of trial frequency (1/36, 2/36, …, 6/36 Hz because of a trial period of 36 s). The results showed that the exponential function fitted well with the grand-averaged ESD of the measured data (goodness of fit, *r*^2^ = 0.99). We postulated that this mathematical form could be applied to individual data, and the same estimation method was used to obtain the parameters of the exponential function best fitted to the ESD data of each region of each participant (averaging the six sessions). In this ESD estimation, we excluded two regions of a single participant (#11) whose ESDs had smaller values at low frequencies than the noises so that the exponential functions could not fit. In the other cases, the exponential functions were successfully fitted to the ESD data of individual regions of participants with *r*^2^ = 0.64 on average, as shown by the examples in Figure 4*B*. The values of parameters *C* and *D* obtained for each region of each participant provided the function:

$$\log\left(\frac{N_{0}^{2}}{{\left|M(f)\right|}^{2}}\right)=-C\exp(-Df),$$

which was used to construct the Wiener filter of Equation 2.

In contrast to M(f) and N(f), which can be estimated using the measured data, we do not know the particular H(f) of the concerned regions in most cases, so that we need to presume a certain form of HRF. Whereas the magnitude of convolution h(t)∗s(t) is constrained by the measured BOLD data, the absolute magnitude of the HRF is arbitrary because the magnitude of the neural activity is not defined in general. Thus, we cannot compare the magnitudes of the deconvolved signals among different regions with a presumed HRF whose magnitude is arbitrarily given. It should be noted here that effective information provided by the deconvolution method in this study was not magnitude (except comparing conditions within the same region) but temporal profile (shape) of activity. It is also critical to consider the fact that the shapes of observed HRFs, which are obtained as BOLD responses to an impulse neural activity, vary across regions and individuals (Aguirre et al., 1998; Handwerker et al., 2004). Our strategy to overcome this problem of the HRF variability was to estimate the sizes of the variability effects on deconvolution results and to show that the effects can be smaller than the fMRI sampling timescale. As the HRF h(t) used in the Wiener deconvolution, we adopted a typical form of HRF, which is characterized by a positive peak and the postpeak undershoot that returns to the baseline within a proper timescale according to the properties described by many studies. The typical HRF was determined by averaging substantial amount of empirical data of human HRFs with excluding apparently atypical cases such as ones whose undershoots do not return to the baseline. Then the Wiener deconvolution using this typical HRF was applied to empirical HRF data without excluding any atypical or outlier cases to estimate the effects of the HRF variability on the deconvolution results. By courtesy of Daniel Handwerker (National Institute of Mental Health) and his collaborators, we were permitted to use the dataset of BOLD impulse responses presented by Handwerker et al. (2004), which includes 80 samples of the BOLD responses empirically obtained from four different regions [the primary motor cortex (M1), primary visual cortex (V1), FEFs, and supplementary eye fields (SEFs)] of 20 participants. Each sample of the BOLD RT series was scaled to 0 at the initial point and 1 at the maximum. It is shown that a reasonably good fit to the BOLD impulse response is provided by a model function comprising a sum of two γ distribution functions (GDFs; Boynton et al., 1996; Friston et al., 1998), and thus we fitted each sample with this type of model. A GDF was given by $G(t,\alpha ,\theta )=\frac{t^{\alpha -1}e^{-\frac{t}{\theta }}}{\theta^{\alpha }\Gamma (\alpha )}$, where α and θ are shape-determining parameters and Γ(α) is a γ function. We fixed θ to 1 for simplicity and omitted it from the fitting scans. The model function was given by:

$$h(t)=A_{1}G\left(t,\alpha_{1}\right)-A_{2}G\left(t,\alpha_{2}\right),$$

where the first and the second GDFs model the positive peak and the undershoot of the HRF, respectively. In our fitting, the four parameters determining amplitudes ($A_{1}$, $A_{2}$) and shapes ($\alpha_{1}$, $\alpha_{2}$) were scanned with a precision of 0.01, to minimize the sum of squared residuals. Because the peak of a GDF (with θ = 1) is obtained at α−1, we set the initial values of the scan parameters for the first and second GDF using the maximum and minimum peaks of the sample data, respectively. Ten of the 80 samples, whose undershoots did not return to the baselines, had no optimal fits with $\alpha_{2}$ under the maximum scanning limit of 23; therefore, these 10 samples were excluded from the following averaging. Another 8 samples had outliers (apart from the quartiles by 1.5 times the interquartile range) of at least one of the four parameters, so that they were also excluded. The fittings were as good as *r*^2^ = 0.940 on average for the remaining 62 samples, with an improvement from 0.934 for including the 18 excluded samples above. The model functions of the 62 samples were calculated from 0 to 32 s with a precision of 0.1 s, normalized to start at 0 and peak at 1, and averaged at each time point. The average model function was again normalized and fitted by a model function, which provided us the dataset-based empirical HRF with parameters $\alpha_{1}$ = 5.10, $A_{1}$ = 5.21, $\alpha_{2}$ = 11.55, and $A_{2}$ = −1.89 (Fig. 4*C*). We used this empirical HRF to construct the Wiener filter. Relating to H(f), whose ESD ${\left|H(f)\right|}^{2}$ is shown in Figure 4*D*, Equation 2 has the problem of diverging (very large) magnitudes of the filter W(f) at high frequencies, where the magnitude of the denominator H(f) is close to zero. With diverging W(f), even small fluctuations in the measured data M(f) can cause considerable disturbances in the deconvolved signals in Equation 1. To prevent this problem, we introduced a positive term δ to increase the minimum of the denominator from zero, as follows:

(3) $$W(f)=\frac{H*(f)}{{\left|H(f)\right|}^{2}+\delta }\left[1-\frac{N_{0}^{2}}{{\left|M(f)\right|}^{2}}\right],$$

where * denotes a complex conjugate. It should also be noted that δ emphasizes the particular frequency components of W(f), where ${\left|H(f)\right|}^{2}$ = δ because the function *x*/(*x*^2^+δ) has a maximum peak at *x*^2^ = δ. To prevent this distortion, we used δ = 24, which is larger than the maximum ${\left|H(f)\right|}^{2}$, so that the influence of δ becomes monotonic over frequency. In this study, we used Equation 3 as the Wiener filter, whose magnitude |W(f)| depends on frequency as shown in Figure 4*E*.

**Figure 5.**
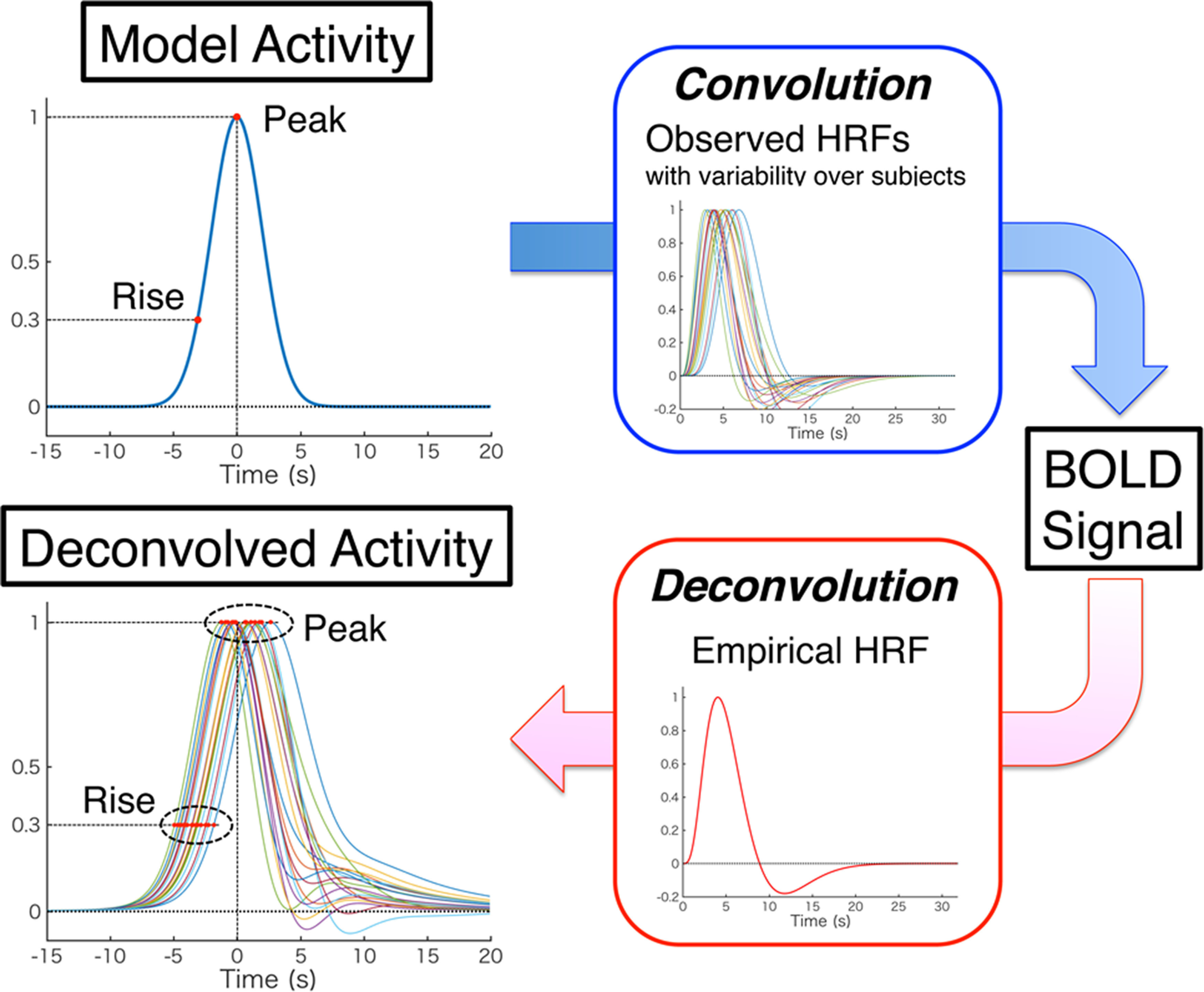
Procedure to estimate precision of the present deconvolution method. A model neural activity was given by a Gaussian function with mean 0 s and SD 2 s, whose peak time and rise time (defined as a time for activity to reach 30% of the amplitude) were 0 and −3.1 s, respectively. The model activity was convolved with 80 HRFs obtained from the dataset of Handwerker et al. (2004) to generate BOLD signals, which reflected the HRF variability over participants and regions. Here, we used all the 80 HRFs of the dataset, including HRFs of the outliers, for the purpose of estimating the effects of HRF variability (the figure displays 20 representative HRFs from the dataset). We did not add noise to the model activity to emphasize influences of HRF variability. These BOLD signals were deconvolved by the Wiener filter Equation 3, which was constructed with the empirical HRF. All the time series of this figure were calculated with the time step of 0.1 s and normalized between 0 at the baseline and 1 at the maximum.

We estimated the influences of the HRF variability over regions and individuals on the activity signals obtained by the deconvolution filter of Equation 3. As shown in Figure 5, we used a model neural activity of a Gaussian function (mean of 0 s and SD of 2 s) and convolved it with the 80 different HRFs of the dataset of Handwerker et al. (2004) to generate BOLD signals. Subsequently, the Wiener deconvolution (Eq. 3) was applied to these BOLD signals to recover the original (model) activity. A two-way ANOVA showed that the deconvolved signals had peak times dependent on both region (*F*_(3,57)_ = 6.60, *p* < 0.001) and individual (*F*_(19,57)_ = 8.03, *p* < 0.001). The peak times of regions averaged over individuals were 0.52 ± 0.86 s (SD) for M1, and −0.16 ± 0.98 s for V1, 0.30 s ± 0.92 s for FEF, and −0.08 ± 0.91 s for SEF. Using these values, we estimated 95% confidence intervals of the mean peak times of the regions averaged over 14 individuals, which were expressed as $mean\pm 1.96\times SD/\sqrt{n}$, where *n* = 14: [0.07, 0.97] (s) for M1, [−0.67, 0.35] for V1, [−0.18, 0.78] for FEF, and [−0.56, 0.40] for SEF. Thus, we could expect to obtain the mean peak times in the bin of the true (model) activity peak time (0 s), which was [−1, 1] with the same width of the sampling time of fMRI (2 s). While the precision of the deconvolved peak time, which is given by the standard error of the mean, can be improved by increasing the number of participants (*n*), the accuracy of the deconvolved peak time of a region, which is the closeness of the mean to the true value, is limited by the properties of HRF intrinsic to the region owing to multiple factors such as vasculature and baseline cerebral blood flow (Handwerker et al., 2004). The estimation above, however, showed that the intrinsic peak-time differences among regions are not significantly large relative to a typical fMRI sampling time (2 s), so that detected differences can be significant in most cases. We also estimated the variability of the rise time of the deconvolved signal, which was defined as the time to reach at 0.3 (30% of the amplitude). The mean difference of rise time from the true (model) value averaged over individuals and regions was −0.76 ± 0.71 s (±SD), indicating that the deconvolved signal rose earlier than the model activity. It seems that this earlier rise was caused by the low-frequency transparency of the Wiener filter (Fig. 4*E*), which is an unavoidable property for the filter to eliminate the high-frequency noise. The above estimations aimed to theoretically show that the differences in the deconvolved signals could reflect significant differences in the original activity despite the HRF variability over individuals and regions. The experimental data, however, include variability caused by many other factors, so that taking a peak of averaged time course could be more effective than averaging peaks taken from time courses, the former of which was the way this study adopted in the following.

### Profile classification of deconvolved signals

**Figure 6.**
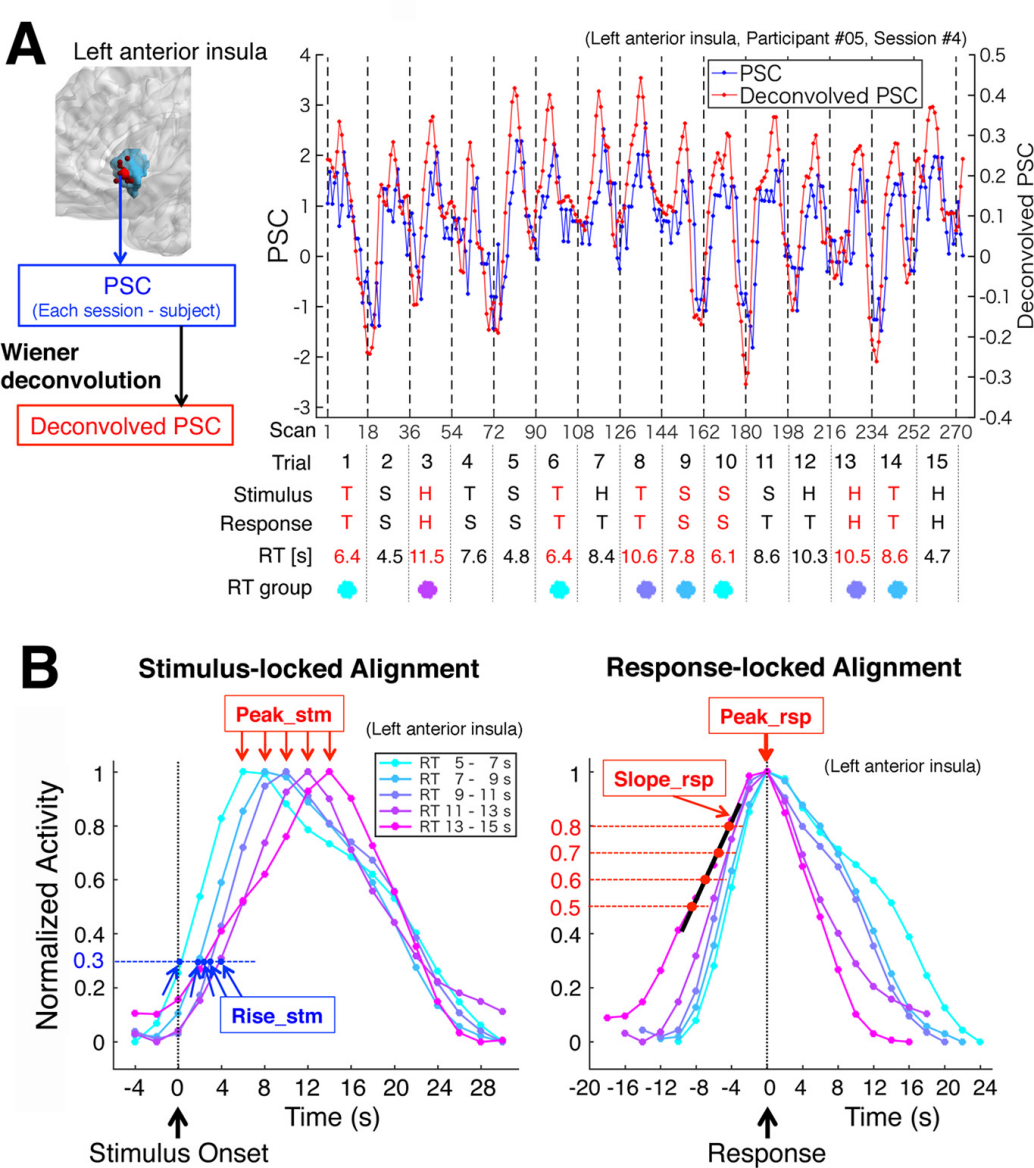
From PSC time series to profile parameters. ***A***, An example of time series of PSC (blue line) and its deconvolved signal (red line) obtained from the left anterior insula of the fourth session of participant #05. The deconvolved signal was segmented in a trial-based manner, and each segment was labeled by stimulus, participant's first answer and RT of the trial. Segments of trials with correct answers and the assigned-range RTs (indicated in red letters) were averaged in the RT groups (indicated by small disks with colors in common with panel ***B***). ***B***, The stimulus-locked averages (left) and the response-locked averages (right) of the deconvolved PSC segments of each RT group across sessions and participants. (For CSRs, the averages were also separated by the region's selectivity to stimuli.) The averaged signals were normalized to have values between 0 and 1 to highlight temporal profiles rather than magnitudes of activity. For classification of activity patterns, we obtained three kinds of parameters to characterize the profiles: Peak_stim and Peak_rsp, which were the peak times of time courses of the two alignments, and Slope_rsp, which was the slope of the line fitted to four points (at signal values of 0.5, 0.6, 0.7, and 0.8) on the rising side of response-locked time course. The black thick line segment indicates the fitted line as an example. We also obtained Rise_stm, which was the time of the rising side of a stimulus-locked time course reached at a signal value of 0.3, to use not for classification but for rationalizing the classification in Results.

As illustrated in Figure 6*A*, a time series of the PSC of a session of the decision section was extracted from each ROI voxel of each participant, and was deconvolved by the Wiener filter. From the deconvolved time series, we extracted segments of trials in which the participant's first responses were correct (68% of all the trials as described above) to avoid intermingling activity of the second-decision process. To evaluate the temporal profiles of activity, the trial segments of each region were averaged separately for participant's RTs of the trials because the activity dependence on decision moment is a critical property of the accumulator regions, and the RT approximates the decision moment. The trial segments were grouped by RT of the trial, with intervals of [5, 7), [7, 9), [9, 11), [11, 13), and [13, 15) (s). (The notation […) indicates an interval with a closed low end and open high end.) We did not use trials with RTs shorter than 5 s or longer than 15 s to avoid high error rates and low observed numbers (see Results). The RT-grouped trial segments were averaged across sessions and participants in both stimulus-locked and response-locked alignments, which were defined by the signal acquisition times relative to the stimulus onset and the participant's response, respectively (Fig. 6*B*). Signal acquisition times were obtained using the slice-timing modeling. In this method, the original slice position of the ROI voxel before the spatial normalization of the image was recovered by reading the normalization parameter file (y_*.nii) produced by SPM12, and the accurate (modeled) slice acquisition time of the voxel was calculated based on the mean relative position of the original slice in scans. For averaging the trial segments, we used time coordinates with 18 bins with a 2-s width (corresponding to the length of a trial) whose centers were multiples of the TR (2 s). The 18 data points of each trial segment were assigned to the bins according to their acquisition times relative to either the stimulus onset or the response. In the stimulus-locked alignment, the bins for all the ROI voxels were made completely matched the scans because the stimulus onset was at the midpoint of the third scan of the trial (as described above); thus, each of the 18 time bins whose centers were −4, −2, 0, …, and 30 s covered acquisition times of all the ROI voxels within a scan relative to the stimulus onset (Fig. 6*B*, left panel). In the response-locked alignment, the 18 time bins were made to have their centers at −4−RT_0_, −2−RT_0_, −RT_0_, …, 0, …, 28−RT_0_, and 30−RT_0_ (s) for trials of an RT group with center RT_0_ (s), in a manner of stimulus-locked coordinates shifted by RT_0_ (Fig. 6*B*, right panel). However, the assignments of data points to the bins were not the same as the simply-RT_0_-shifted stimulus-locked assignments because the response-locked time bins depended on both the acquisition times of data points and RT of the trial. For example, consider a trial with an RT of 7.8 s, which is grouped into RT_0_ of 8 s. The bin of 0 s of the response-locked alignment can include data points with acquisition times ranging from RT − 1 (= 6.8 s) to RT + 1 (= 8.8 s), which can be assigned to bins of 6 and 8 s of the stimulus-locked alignment. By a similar trace, in the case of RT 8.2 s of the same group of RT_0_ 8 s, data points of the same bin of 0 s of the response-locked alignment can be assigned to bins of 8 s and 10 s of the stimulus-locked alignment. Generally, a bin of the response-locked alignment can include data points from three consecutive stimulus-locked bins, reflecting temporal relations between the signal acquisitions of the voxels and the response moments more precisely than the simply-RT_0_-shifted stimulus-locked alignment. Therefore, employing both the two kinds of alignments seems to be effective to obtain accurate properties of activity profiles. The averaged signal segments were normalized to have values between 0 and 1, as shown in Figure 6*B*. We refer to this “normalized average deconvolved PSC” as “normalized activity” for short, because the deconvolved PSC is the estimated neural activity.

To classify the activity profiles of regions, we basically followed the approach of Ploran et al. (2007), whereas we also took a heuristic approach when it was necessary. We initially postulated peak and rise times of activity signals as the parameters to characterize the activity profiles because the region classes of Ploran et al. (2007) were defined by dependence of these parameters on the decision moment: both of the two parameters show the dependence in the moment-of-recognition regions, only the peak time in the accumulators, and neither in the sensory processors. We also evaluated validity of the present method by reproducibility of the classification results of Ploran et al. (2007, 2011), the latter of which was an improved version of the former study showing substantially similar classification results with modified methods of stimulation and clustering. [In this study, our approach basically followed Ploran et al. (2007), while we referred to the results of Ploran et al. (2011) as well.] In a heuristic manner, we found that the rising slope was more effective for reproducing the classification results of Ploran et al. (2007, 2011) than the rise time as described in Results, so that we used the rising slope of the response-locked time course (referred to as Slope_rsp) instead of the rise time. The three profile parameters, which were the peak times of the stimulus-locked and response-locked time courses (referred to as Peak_stm and Peak_rsp, respectively) and Slope_rsp, were extracted from each of the RT-group-averaged signal segments, as shown in Figure 6*B*. For Slope_rsp, four points on the rising side of each response-locked time course were obtained at heights of 0.5, 0.6, 0.7, and 0.8 by interpolating between the consecutive sampling points that sandwiched these heights, and Slope_rsp was defined as the slope of the line fitted to these four points. As shown in Figure 6*B*, the rise time of the stimulus-locked time course (Rise_stm) was defined as the time of the rising side of the time course at a height of 0.3, which was obtained by interpolating between the consecutive sampling points that sandwiched 0.3 in height. Rise_stm was not used for the classification but to rationalize the present method. To reproduce similar results as the classification of Ploran et al. (2007, 2011), we used four statistical quantities of the profile parameters over RTs as follows: SD of Peak_stm (Peak_stm_SD), SD of Peak_rsp (Peak_rsp_SD), mean of Slope_rsp (Slope_rsp_MN), and mean of Peak_rsp (Peak_rsp_MN). The first three quantities were used to reflect the different dependence on decision moment (i.e., RT), while the fourth was employed in a heuristic manner as described in Results.

We classified the regions by means of a hierarchical cluster analysis in the four-dimensional space of the above four quantities using Ward's method (Ward, 1963) following Ploran et al. (2007). In this method, at the initial step, each data point is regarded as a cluster comprising one member; in the next step, a new cluster is created by merging a pair of clusters that minimizes the increase in the sum of the squared Euclidean distances of all the member data points of the new cluster from their centroid (average coordinates). This cluster-merging step is repeated until all data points are included in one cluster to form a clustering tree (dendrogram) that represents the proximity among data points in a hierarchical manner. Because Ward's method uses the Euclidean distances in the multidimensional space of the parameters, the clustering results can be affected by the absolute scales of the parameters. To evaluate the parameters with equal weights, we scaled the SD of the values of each parameter over the regions to 1 by dividing by their original SD before applying Ward's method. In this study, we used Ward's algorithm implemented in MATLAB Statistics and Machine Learning Toolbox (version 11.0; function linkage).

In the data analyses of this study, we used applications including SPM12 and homemade programs working on MATLAB 2016b. Statistical tests were performed using SPSS version 21 (IBM Corp.) and G*Power (Faul et al., 2007). Visualization of brain mapping in Figures 3, 6, 12 was conducted with the BrainNet Viewer (version 1.53; Xia et al., 2013; http://www.nitrc.org/projects/bnv/).

## Results

### Behavioral results

**Figure 7.**
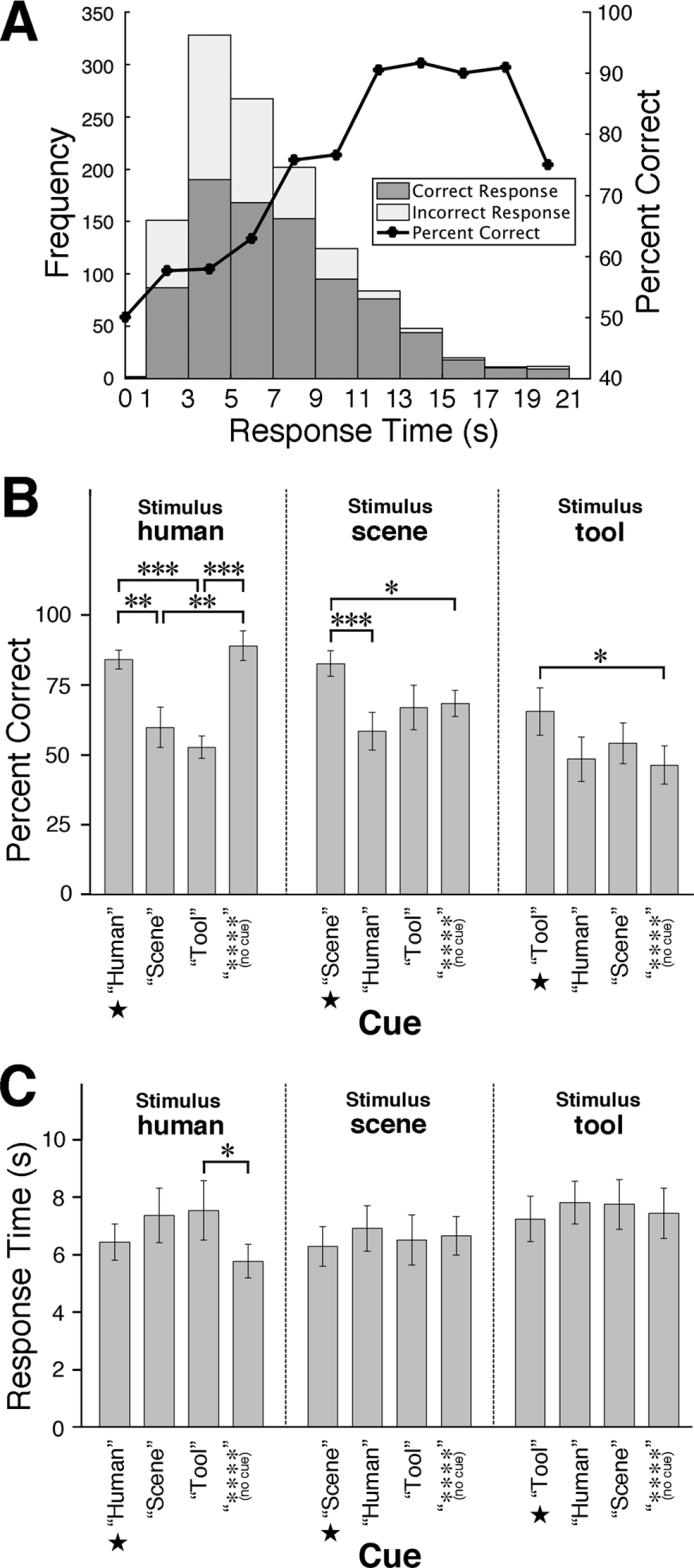
Behavioral performance of the categorization task in the decision section, regarding the first responses of trials. ***A***, Left axis, Histogram of response observations on RT of all the trials over stimuli and participants. Right axis, Relationship between accuracy (percent correct) and RT. ***B***, Accuracy averaged across participants. Cues congruent with the stimuli are denoted by ★. In each stimulus condition, multiple comparisons of the simple effects of cues were conducted with Bonferroni correction: *p < 0.05, **p < 0.01, ***p < 0.001. Error bars show SEM. ***C***, Mean RT averaged across participants. The indications are the same as ***B***.

In the decision section, while participants were allowed to make second or later responses to revise their answers, we used only trials of the correct first responses, which were 68% of all trials across participants, in the fMRI analysis to avoid intermingling the second-decision process. Figure 7*A* shows the histogram of the first RT, and the relationship between accuracy (percent correct) and RT. The accuracy was higher with longer RT (except for the highest RTs with low observation frequencies), which seems to be consistent with the general property of the accumulation model using various decision thresholds pooled over participants (Bogacz et al., 2006). A cue word was given immediately before each stimulus epoch to determine the influence of prior knowledge on accumulation activity. We expected that a stimulus-congruent cue would facilitate decision while a stimulus-incongruent cue would obstruct the decision to the contrary. The results showed that the effects were not as simple as the expectation. A 3×4 repeated measures ANOVA of accuracy with factors of stimulus (human, scene, and tool) and cue [“Human,” “Scene,” “Tool,” and “****” (no cue)], passing Mauchly's test of sphericity (*p* > 0.05), showed significant effects of stimulus (*F*_(2,26)_ = 11.08, *p* < 0.001) and of the interaction of cue and stimulus (*F*_(6,78)_ = 8.44, *p* < 0.001) but not of cue (*F*_(3,39)_ = 1.16, *p* = 0.34). Multiple comparisons with Bonferroni correction of the simple effects of cues showed only the obstruction by the incongruent cues for human stimulus, while only the facilitation effects of congruent cues were observed for scene and tool stimuli (Fig. 7*B*), indicating that the cue congruency effects varied depending on the stimulus. The same statistical tests were applied to cue congruency effects on RT, and the repeated measures ANOVA of RT showed significant effects of stimulus (*F*_(2,26)_ = 18.81, *p* < 0.001) and the interaction of cue and stimulus (*F*_(6,78)_ = 4.26, *p* < 0.001) but not of cue (*F*_(3,39)_ = 2.38, *p* = 0.084). Multiple comparisons showed that cue congruency did not have a significant effect on RT in most of the comparisons (Fig. 7*C*). In these results, the cue congruency effects were more complicated and less obvious than expected. Therefore, we pooled the cue conditions in the following analyses. As a possible reason for the less notable effects of cue congruency, participants noticed a substantial proportion of cues being incongruent early in the experiment and did not rely on the cues to make decisions, which was reported spontaneously by more than half of the participants in the postexperiment debriefing.

### Functional localization of CSRs

We determined CSRs using fMRI data of the localization section, and the coordinates of their peak voxels were selected by means of the “individual peaks within group activation” method (see Materials and Methods). First, we conducted group-level analyses of SPM for the four kinds of category selectivity (face, body, scene, and tool) by applying the inclusive mask of positive activation, and 14 ROIs of clusters of contiguous voxels were detected at a 0.1% voxel-wise threshold and a cluster-wise threshold of the FWER 5%. Second, applying each of these ROIs as an inclusive mask, we detected the peak voxels in the ROIs of the corresponding categories in the individual-level analysis. We excluded six of the 14 ROIs in which two or more participants showed no significant voxels, and the remaining eight ROIs were identified as CSRs, which were two right face-selective regions (referred to as R Face-SR 1 and R Face-SR 2), one left body-selective region (L Body-SR), one right body-selective region (R Body-SR), two left scene-selective regions (L Scene-SR 1 and L Scene-SR 2), one right scene-selective region (R Scene-SR), and one left tool-selective region (L Tool-SR). Finally, we selected the peak voxel showing the strongest effect in each of the eight CSRs of each participant, with diminishing spatial dispersion of the voxels among participants as described in Materials and Methods. The location of each CSR was represented by the mean coordinates among the participants of that ROI. Table 1 shows that the coordinates of these CSRs, which, except L Scene-SR 2, were close to the CSRs identified in the previous studies. Based on this correspondence, we can reasonably regard R Face-SR 1 as the right fusiform face area (Kanwisher et al., 1997), R Face-SR 2 as the right occipital face area (Kovács et al., 2008), bilateral Body-SRs as the extrastriate body areas (Downing et al., 2001), L Scene-SR 1 and R Scene-SR as the posterior extension of the parahippocampal place areas (Epstein and Kanwisher, 1998), and L Tool-SR as the lateral occipital complex (Grill-Spector et al., 1999). The peculiarity of L Scene-SR 2 is discussed below.

**Table 1.** Locations of CSRs

										Previous studies				
ROI idx.	Region name	*x* (mm)	*y*	*z*	Anatomical location	BA	Region vol. (voxels)	Number of partic.	SD of ind. coord. (mm)	Lit.	*x* (mm)	*y*	*z*	Distance (mm)
(1)	R Face-SR 1	44	−54	−21	R fusiform gyrus	37	112	14	4.8	(1)	40	−57	−15	7.8
(2)	R Face-SR 2	50	−70	−2	R inferior occipital gyrus	37	210	14	7.2	(2)	47	−73	−12	10.9
(3)	L Body-SR	−52	−70	5	L inferior occipital gyrus	37	588	14	7.4	(3)	−51	−72	8	3.7
(4)	R Body-SR	52	−67	1	R inferior occipital gyrus	37	964	14	8.0	(3)	51	−71	1	4.1
(5)	L Scene-SR 1	−28	−45	−11	L fusiform gyrus	37	356	14	5.9	(4)	−34	−31	−9	15.4
(6)	L Scene-SR 2	−12	−90	−5	L calcarine cortex	18	161	13	7.2					
(7)	R Scene-SR	30	−45	−11	R fusiform gyrus	37	445	13	9.0	(4)	18	−40	−9	13.2
(8)	L Tool-SR	−40	−67	−11	L inferior occipital gyrus	19	921	13	13.2	(5)	−36	−74	−19	11.4

ROI idx., ROI index. *x*, *y*, *z*, the mean MNI coordinates of all participants of the ROI. Anatomical location (tissue labeling) of the mean coordinates was provided by TPM of SPM12 (when the label was the white matter, that of a neighboring voxel was used). BA, Brodmann's area of the mean coordinates provided by MRIcron. Region vol., region volume in voxels of activation in group-level analysis. Number of partic., the number of participants who showed significant voxels within the ROI. SD of ind. coord., SD (in 3D space) of MNI coordinates of individual participants, the smaller value suggesting the more cohesive anatomic locations among participants. Previous studies, representative reports of CSR locations (in MNI coordinates): (1) Kanwisher et al. (1997), (2) Kovács et al. (2008), (3) Downing et al. (2001), (4) Epstein and Kanwisher (1998), and (5) Grill-Spector et al. (1999). Distance, Euclid distance between the coordinates of the present and previous studies. In the column of region name: L, left. R, right. -SR, -selective region. Full descriptions of the abbreviations are in the main text.

### Localization of NCSRs

The NCSRs were defined as the TPM-based regions that showed the decision task-related positive activation without any significant category selectivity. We used the volume data of 121 atlas regions of the TPM as anatomic masks to parcel the noncategory-selective range. Applying each TPM-atlas mask together with the masks of positive activation and noncategory selectivity inclusively to the individual-level *t*-contrast image of the task-positive activation, in the same manner as CSRs we extracted the peak voxel showing the strongest task effect in the individual-level analysis of each participant, with diminishing spatial dispersion of the voxels among participants. We adopted 55 NCSRs, in which 14 or 13 participants had significant voxels in the individual-level analyses (Table 2).

**Table 2.** Locations of NCSRs

ROI idx.	Region name	*x* (mm)	*y*	*z*	BA	Number of partic.	SD of ind. coord. (mm)
(9)	L AIns (anterior insula)	−29	22	−2	47	14	4.8
(10)	R AIns (anterior insula)	32	24	1	47	14	5.8
(11)	L AnG (angular gyrus)	−27	−65	43	7	14	5.4
(12)	L Caudate	−14	−1	15	NA	14	5.1
(13)	R Caudate	16	0	16	NA	13	5.0
(14)	L Cerebellum Exterior	−35	−72	−24	NA	14	12.0
(15)	R Cerebellum Exterior	35	−65	−26	NA	14	14.5
(16)	L FO (frontal operculum)	−33	19	7	48	14	6.5
(17)	R FO (frontal operculum)	36	23	6	48	14	5.0
(18)	L FuG (fusiform gyrus)	−32	−61	−14	19	14	1.0
(19)	R FuG (fusiform gyrus)	37	−42	−19	37	14	6.9
(20)	L Hippocampus	−19	−32	−6	NA	14	3.6
(21)	R Hippocampus	21	−30	−5	NA	14	3.1
(22)	L IOG (inferior occipital gyrus)	−27	−90	0	18	14	4.0
(23)	R IOG (inferior occipital gyrus)	32	−89	5	18	14	2.4
(24)	L ITG (inferior temporal gyrus)	−46	−52	−12	37	14	3.3
(25)	R ITG (inferior temporal gyrus)	44	−50	−10	37	14	0.9
(26)	R MCgG (middle cingulate gyrus)	6	18	37	24	14	4.8
(27)	L MFG (middle frontal gyrus)	−39	7	31	44	14	6.0
(28)	R MFG (middle frontal gyrus)	38	5	42	6	14	12.0
(29)	L MOG (middle occipital gyrus)	−28	−92	8	18	14	1.8
(30)	R MOG (middle occipital gyrus)	32	−84	12	18	14	0.0
(31)	L MSFG (superior frontal gyrus medial segment)	−4	27	42	32	14	4.7
(32)	R MSFG (superior frontal gyrus medial segment)	7	27	37	32	14	3.8
(33)	L OCP (occipital pole)	−22	−98	6	17	14	0.0
(34)	L OFuG (occipital fusiform gyrus)	−26	−87	−11	18	14	2.9
(35)	R OFuG (occipital fusiform gyrus)	29	−86	−11	18	14	2.5
(36)	L OpIFG (opercular part of the inferior frontal gyrus)	−41	11	25	48	14	6.7
(37)	R OpIFG (opercular part of the inferior frontal gyrus)	47	12	30	44	14	2.8
(38)	L OrIFG (orbital part of the inferior frontal gyrus)	−30	27	−1	47	14	3.7
(39)	R OrIFG (orbital part of the inferior frontal gyrus)	34	28	−1	47	14	3.8
(40)	L Pallidum	−17	0	1	NA	14	6.1
(41)	R Pallidum	18	5	0	NA	14	6.3
(42)	L PHG (parahippocampal gyrus)	−16	−33	−5	27	14	2.5
(43)	R PHG (parahippocampal gyrus)	18	−32	−6	27	14	2.5
(44)	L PrG (precentral gyrus)	−43	5	31	44	14	6.0
(45)	R PrG (precentral gyrus)	46	5	33	44	14	8.4
(46)	L Putamen	−22	3	−2	NA	14	8.5
(47)	R Putamen	23	7	−1	NA	14	9.8
(48)	R SFG (superior frontal gyrus)	25	4	59	6	14	9.3
(49)	L SMC (supplementary motor cortex)	−4	9	52	6	14	5.5
(50)	R SMC (supplementary motor cortex)	7	14	50	32	14	8.0
(51)	R SMG (supramarginal gyrus)	45	−29	42	2	14	3.6
(52)	L SOG (superior occipital gyrus)	−26	−94	11	18	13	1.0
(53)	R SOG (superior occipital gyrus)	30	−84	14	18	14	1.0
(54)	L SPL (superior parietal lobule)	−24	−62	51	7	14	6.5
(55)	R SPL (superior parietal lobule)	24	−60	53	7	14	5.2
(56)	L Thalamus Proper	−19	−31	−4	NA	14	3.3
(57)	R Thalamus Proper	22	−29	−3	NA	14	3.0
(58)	L TrIFG (triangular part of the inferior frontal gyrus)	−39	30	14	48	14	5.4
(59)	R TrIFG (triangular part of the inferior frontal gyrus)	49	35	14	45	14	6.5
(60)	L Ventral DC (diencephalon)	−19	−26	−6	NA	14	6.8
(61)	R Ventral DC (diencephalon)	20	−25	−6	NA	14	6.3
(62)	Cerebellar Vermal Lobules VI-VII	−2	−76	−31	NA	14	6.1
(63)	Cerebellar Vermal Lobules VIII-X	0	−55	−37	NA	13	3.3

The order and abbreviations of the regions followed the TPM database of SPM12. Abbreviations of the column headings are the same as Table 1. NA, not applicable.

We examined the activity dependence of the CSRs and NCSRs on stimulus category in the decision section because it was not obvious how their category selectivity, which was determined by using the easy-to-categorize stimuli in the localization section, would be kept in response to degraded difficult-to-categorize stimuli of the decision section. A blocked design SPM analysis was applied to the BOLD data of the decision section (preprocessed with the slice timing correction) using models of boxcar-shaped activation during the stimulus epochs of three categories (human, scene, and tool), which were confined to trials of the participant's correct first responses. We extracted the β values (regression coefficients of the models on BOLD signals over six sessions) from the contrast images of stimulus conditions at the coordinates of CSRs and NCSRs of each participant. These β values were analyzed using repeated measures ANOVA with a factor of stimulus (human, scene, and tool). Table 3 shows that the effects of stimulus were highly significant (*p* < 0.001 or 0.01) for all the CSRs except L Scene-SR 2, while significant effects were found in only 9 of 55 NCSRs, most of whose significance levels were not high (*p* < 0.05). Each CSR except L Scene-SR 2 showed that the β of its preferred stimulus was significantly larger than those of both the other two nonpreferred stimuli, which is consistent with the definition of category selectivity employed in the previous studies (see Materials and Methods). On the other hand, none of the NCSRs had its preferred stimulus that took precedence over both the other two stimuli. The results justified averaging the CSR signals of the decision section separately for preferred and nonpreferred stimuli because the signals were significantly different depending on the preference of stimuli.

**Table 3.** Dependence of activation on stimulus in the decision section

	ROI idx.	Region name	rm ANOVA	Human > scene	Human > tool	Scene > human	Scene > tool	Tool > human	Tool > scene
CSR	(1)	R Face-SR 1	***	***	***				
	(2)	R Face-SR 2	***	***	***				
	(3)	L Body-SR	***	***	***				*
	(4)	R Body-SR	***	***	**				**
	(5)	L Scene-SR 1	***			***	**		
	(6)	L Scene-SR 2							
	(7)	R Scene-SR	***			**	**		
	(8)	L Tool-SR	**					*	*
NCSR	(11)	L AnG	*						*
	(18)	L FuG	***			**		*	
	(24)	L ITG	*						*
	(27)	L MFG	*						
	(32)	R MSFG	*						
	(44)	L PrG	*						*
	(49)	L SMC	*						
	(50)	R SMC	*						
	(63)	Cerebellar Vermal Lobules VIII-X	*						*

rm ANOVA, repeated measures ANOVA of β values regarding the stimulus conditions (human, scene, and tool). The ANOVA is followed by multiple comparisons with Bonferroni correction between the stimulus conditions.

*p < 0.05,

**p < 0.01,

***p < 0.001. For NCSRs, only regions that showed significant effects of stimulus conditions are shown. Six of 882 cases in total (63 regions × 14 participants) did not have their coordinates owing to the absence of significant voxels, and we used the coordinates averaged over other participants to fill the data blanks of ANOVA. Multiple comparisons were two-sided. Take notice that all the CSRs except L Scene-SR 2 showed the precedence of their preferential stimuli over both of the other two stimuli.

Regarding the peculiarity of L Scene-SR 2, whose category selectivity was inconsistent between the localization and decision sections, we investigated the stimulus images used in the localization section by spatial frequency analysis, and found that the scene images contained markedly more high-frequency components than images of the other categories. This difference seems to originate from the image components; the backgrounds of the scene images were not removed to keep the expanse of scenery as the content, while those of the other-category images were removed and filled with uniform gray (Fig. 1*B*). Thus, the scene images included more lines and edges, which contributed to the high-frequency components and could strongly activate the early visual areas such as the calcarine cortex where L Scene-SR 2 was identified (Table 1). This would not be the case in the decision sections in which all the stimuli kept their background of the objects. Because L Scene-SR 2 seems not to represent the high-level content of the scene category, we excluded it from the following analysis of CSRs.

### Classification of NCSRs based on activity profiles

**Figure 8.**
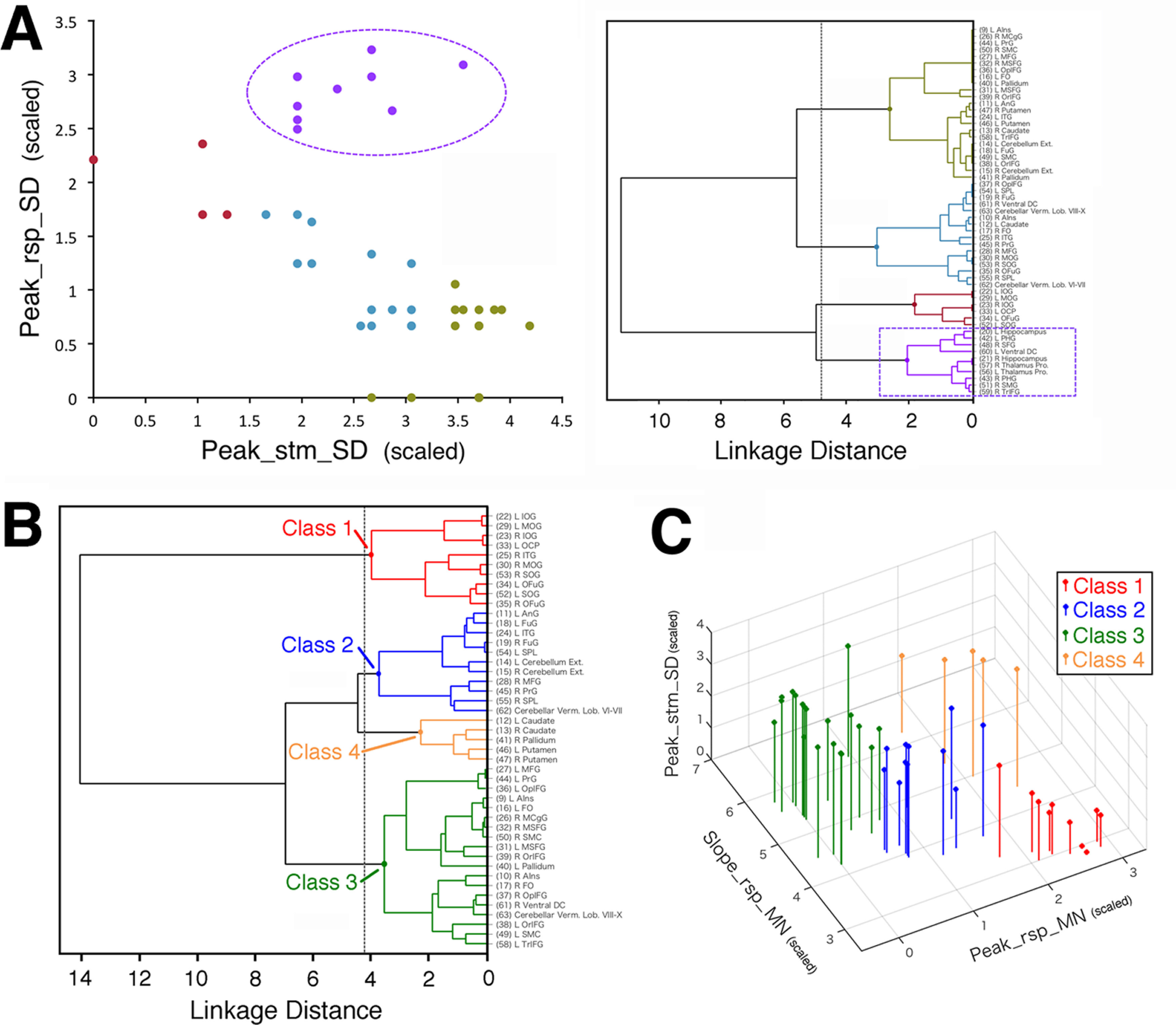
Classification of NCSRs based on the profile-parameter statistics. ***A***, Left panel, A scatter plot between Peak_stm_SD (the SD of the stimulus-locked peak times over RT groups) and Peak_rsp_SD (the SD of the response-locked peak times over RT groups) of the 55 NCSRs. To prevent unequal weights of the parameters caused by their different scales from biasing the following Ward's clustering, each of these parameters was scaled so that the SD over regions was 1, being indicated by the label “(scaled)” of the axis (see Materials and Methods). The 10 regions surrounded by a dashed circle colored violet had large values of both Peak_stm_SD and Peak_rsp_SD, being distinctively apart from the other regions. Right panel, A dendrogram of the 55 NCSRs obtained by Ward's clustering method in the two-dimensional parameter space described above. By cutting the dendrogram at a linkage distance of 4.8, we obtained the four distinct clusters, one of which comprised the same 10 regions as the left panel, shown by the surrounding dashed lines colored violet. (See Table 2 for the abbreviations of the region names.) ***B***, A dendrogram of the 45 NCSRs obtained by Ward's clustering method in the four-dimensional parameter space comprising Peak_stm_SD, Peak_rsp_SD, Slope_rsp_MN (the mean of rising slopes of the response-locked time courses over the RT groups), and Peak_rsp_MN (the mean of the response-locked peak times over RT groups). Four meaningful clusters (indicated as Class 1–4) were generated by cutting the dendrogram at a linkage distance of 4.2, which was determined based on the inspection of the parameter values (see the text). ***C***, A scatter plot of the parameter values in the three-dimensional space of Peak_stm_SD, Slope_rsp_MN, and Peak_rsp_MN to illustrate segregation of the classes (the clustering was conducted in the four-dimensional space with the addition of Peak_rsp_SD). Each of these parameters was scaled to have the SD over regions being 1 as in panel ***A***.

As described in Materials and Methods, time series of BOLD signals were extracted from the fMRI data of the decision section at the coordinates of CSRs and NCSRs determined for each participant, and deconvolved by means of the Wiener filter. Trial segments of the deconvolved signals were averaged separately for the regions and the RT-groups of the trials in both the stimulus-locked and response-locked alignments, and the temporal-profile parameters such as Peak_stm (peak time of the stimulus-locked time course) were extracted from the averaged trial-segment time courses. The statistics of the profile parameters across RTs were used to classify the regions as follows. We first investigated the NCSRs to establish a method of classifying the activity profiles of decision-related regions, and then examined the CSRs using this classification. Because our primary aim was to clarify relations among multiple accumulators and other decision-related regions such as those shown by Ploran et al. (2007, 2011), we confined our investigation to regions whose activity peaks were either response-dependent or stimulus-dependent. For this restriction, we evaluated the Peak_rsp_SD and Peak_stm_SD (SDs of the response-locked and stimulus-locked peak times over RT groups), which reflected temporal cohesiveness (i.e., the degree of timing constancy) of the peaks relative to the response and the stimulus onset, respectively. Figure 8*A*, left panel, shows a scatter plot between the Peak_stm_SD and Peak_rsp_SD of the 55 NCSRs, in which the 10 regions colored violet had large values of both the Peak_stm_SD and Peak_rsp_SD, being distinctively apart from the other regions. The right panel confirms this observation by Ward's clustering method applied to this two-dimensional space, which generated a dendrogram showing these 10 regions as a different cluster from the other regions. These 10 regions were neither response dependent nor stimulus dependent; therefore, we excluded them from further analysis of classification. To classify the remaining 45 NCSRs, we initially used the SD of the rise times over RTs (in either stimulus-locked or response-locked alignments) as well as the Peak_stm_SD and Peak_rsp_SD for separating the regions based on the peak properties hypothesized by Ploran et al. (2007). In our results, however, Ward's clustering in the three-dimensional space comprising these parameters could not provide satisfactory reproduction of the results of Ploran et al. (2007, 2011), especially of separation between accumulators and moment-of-recognition regions. In a heuristic manner, we found that this separation was remarkably improved by employing the mean of rising slopes of the response-locked time courses over the RT groups (Slope_rsp_MN) instead of the SD of rise times. (Possible reasons for this empirical improvement are discussed below.) We also found that there was substantial dispersion of the mean of the response-locked peak times over RT groups (Peak_rsp_MN) among the regions classified as accumulator and moment-of-recognition region in our results. Thus, we added Peak_rsp_MN as the fourth parameter to the clustering space. Then, in the 4-D space of Peak_stm_SD, Peak_rsp_SD, Peak_rsp_MN, and Slope_rsp_MN, Ward's clustering generated a dendrogram of the 45 regions, as shown in Figure 8*B*. The dendrogram at a linkage distance of 4.2 provided the four classes of the regions, which reasonably reproduced the results of Ploran et al. (2007, 2011) with some necessary modifications as described below. Figure 8*C* also shows that the classes were clearly segregated in this parameter space.

**Figure 9.**
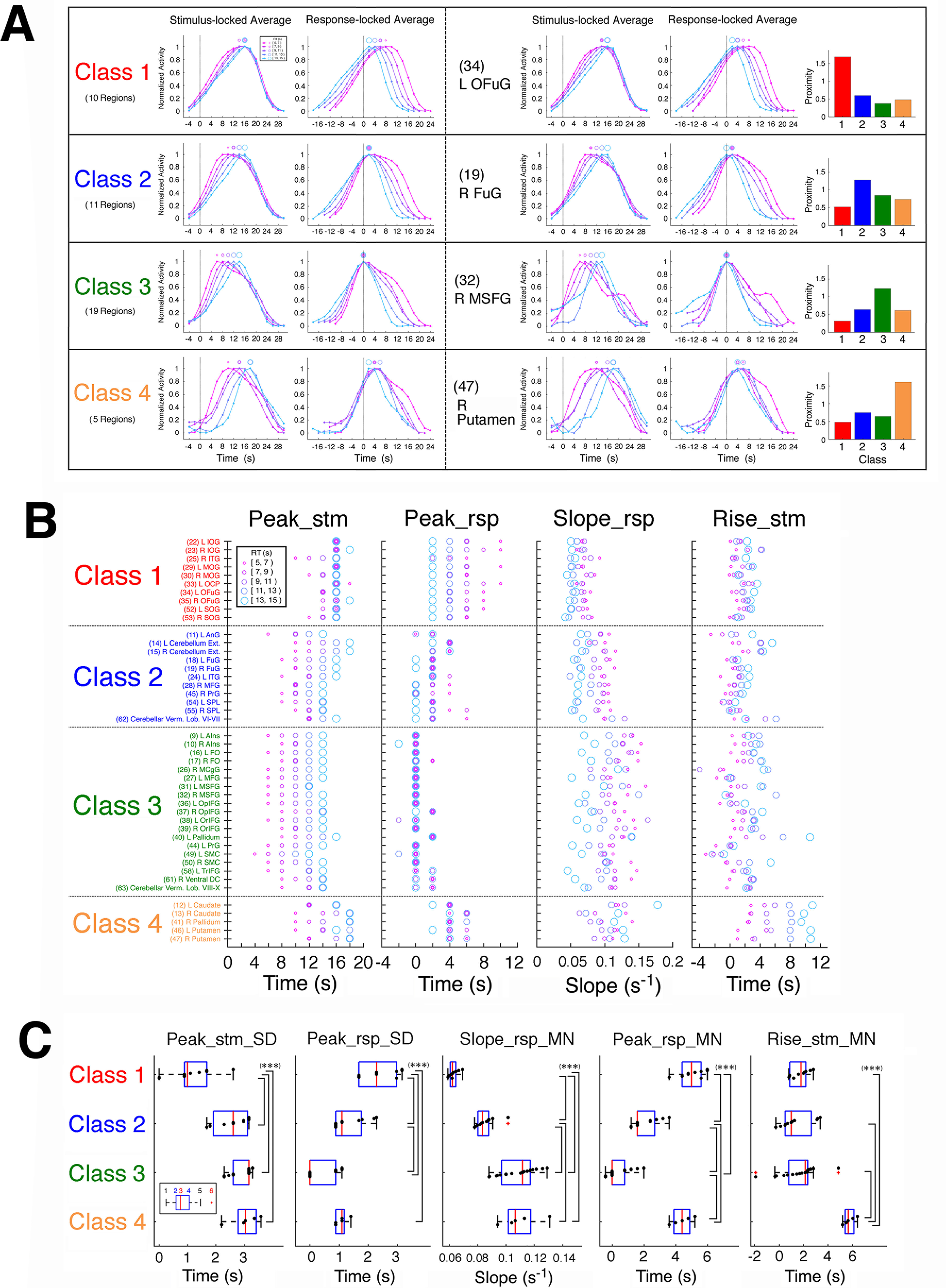
Activity profiles of NCSRs. ***A***, Activity time courses of the averages over the regions of each class (left panels) and those of typical regions selected for each class (right panels). (See Table 2 for the list of the regions.) The origins of time coordinates are stimulus onset and participant's response in the stimulus-locked average and the response-locked average, respectively. For selecting typical regions of the classes in the right panels, we estimated “proximity” of each region to the NCSR classes. Based on the distance *r* between the coordinates of the region and the mean coordinates (the center) averaged over all the regions of each of the four classes in the four-dimensional clustering space of the profile parameters, the proximity was defined as a quantity 1−log r, which indicates how close the region was to the center of the class (shown in the rightmost figures). A region with the largest proximity to each class was selected as the typical region of the class. ***B***, The profile parameters of the individual NCSRs obtained for each of RTs. ***C***, Box plots of the profile-parameter statistics (i.e., mean, SD) across RTs for the NCSRs. The data points represent the statistical values of regions and the box plots represent their distribution for each of the classes. The inset displays the elements of a box plot: 1–6, respectively, indicate minimum, lower quartile, median, upper quartile, maximum, and outlier. The outliers were defined as values larger than the upper quartile or smaller than the lower quartile by 1.5 times the interquartile range. The minimum and maximum were taken excluding the outliers. Data points were overlaid as black dots on the box plots. Multiple comparisons with Bonferonni correction showed significant differences among the classes. The significance level was *p* < 0.001 (denoted as ***) for all differences detected here. While the parameters of the first four panels were used for the clustering of regions, the parameter Rise_stm_MN of the fifth panel was not used for the clustering but was essential for interpreting the new results of classification (see the main text).

Figure 9*A* shows activity profiles of the averages over the regions and of typical regions for the four classes. Evidently, these classes showed different activity profiles regarding the peak and rise times. The profile differences in the averages could mostly be observed in the typical regions, too. These observations are confirmed by the noticeably different distributions of the profile parameters of individual regions, which are Peak_stm, Peak_rsp, Slope_rsp, and Rise_stm, across the classes as shown in Figure 9*B*. Figure 9*C* shows that the four classes were characterized by statistical properties of the profile parameter values as follows. In the first two panels, Class 1 showed significantly smaller Peak_stm_SD and larger Peak_rsp_SD than the other three classes, indicating that Class 1 had the stimulus onset-dependent peak times. In contrast, the peak times of Classes 2, 3, and four were dependent on RTs because of their small Peak_rsp_SDs and large Peak_stm_SDs. In the third panel, Slope_rsp_MNs of both Class 3 and Class 4 were significantly greater than those of Classes 1 and 2. Based on these properties, we can see the following correspondence of classes between the present results and Ploran et al. (2007, 2011): Class 1 to sensory processor, Class 2 to accumulator, and both Class 3 and Class 4 to moment-of-recognition regions, based on the hypothesis by Ploran et al. (2007) from which the slopes of the scaled activity are expected to be steeper in the moment-of-recognition regions than in the accumulators. Table 4 shows the consistency of this correspondence in the anatomic relations, which substantially overlap between the studies.

**Table 4.** Anatomical correspondence between Ploran et al. (2007, 2011) and the present study

The present study					Ploran et al. (2007)						Ploran et al. (2011)					
Class	Region name	*x* (mm)	*y*	*z*	Region name	*x* (mm)	*y*	*z*	Dist.	Class	Region name	*x* (mm)	*y*	*z*	Dist.	Class
Class 1	(22) L IOG	−27	−90	0	L cuneus	−19	−102	−3	14.7	sens	L cuneus	−28	−94	−7	8.1	sens
	(23) R IOG	32	−89	5	R mid occipital G	30	−81	14	12.2	sens	R mid occipital G	31	−90	3	2.4	sens
	(25) R ITG	44	−50	−10	R fusiform G	49	−63	−14	14.5	accum	R fusiform G	44	−65	−11	15.0	IT
	(29) L MOG	−28	−92	8	L mid occipital G	−30	−81	19	15.7	accum	L mid occipital G	−32	−88	7	5.7	sens
	(30) R MOG	32	−84	12	R mid occipital G	30	−81	14	4.1	sens	R mid occipital G	33	−83	16	4.2	accum
	(33) L OCP	−22	−98	6	L cuneus	−19	−102	−3	10.3	sens	L mid occipital G	−32	−88	7	14.2	sens
	(34) L OFuG	−26	−87	−11	L inf occipital G	−32	−92	−16	9.3	accum	L cuneus	−28	−94	−7	8.3	sens
	(35) R OFuG	29	−86	−11					> 20		R inf occipital G	29	−91	−11	5.0	sens
	(52) L SOG	−26	−94	11	L mid occipital G	−30	−81	19	15.8	accum	L mid occipital G	−32	−88	7	9.4	sens
	(53) R SOG	30	−84	14	R mid occipital G	30	−81	14	3.0	sens	R mid occipital G	33	−83	16	3.7	accum
Class 2	(11) L AnG	−27	−65	43	L intraparietal S	−26	−72	38	8.7	accum	L intraparietal S	−24	−61	46	5.8	accum
	(14) L Cerebellum Ext.	−35	−72	−24	L cerebellum	−34	−76	−36	12.7	accum	L fusiform G	−29	−73	−17	9.3	IT
	(15) R Cerebellum Ext.	35	−65	−26	R fusiform G	49	−63	−14	18.5	accum	R fusiform G	33	−58	−23	7.9	IT
	(18) L FuG	−32	−61	−14	L fusiform G	−42	−65	−14	10.8	accum	L fusiform G	−30	−57	−21	8.3	IT
	(19) R FuG	37	−42	−19					> 20		R fusiform G	28	−48	−22	11.2	IT
	(24) L ITG	−46	−52	−12	L fusiform G	−42	−65	−14	13.7	accum	L fusiform G	−40	−67	−17	16.9	IT
	(28) R MFG	38	5	42	R inf frontal G	44	4	36	8.5	accum	R inf frontal G	39	4	31	11.1	accum
	(45) R PrG	46	5	33	R inf frontal G	44	4	36	3.7	accum	R inf frontal G	39	4	31	7.3	accum
	(54) L SPL	−24	−62	51	L intraparietal S	−26	−72	38	16.5	accum	L intraparietal S	−24	−61	46	5.1	accum
	(55) R SPL	24	−60	53	R inf parietal lobule	34	−61	48	11.2	recog	R intraparietal S	27	−59	46	7.7	accum
	(62) Cerebellar Verm. Lob. VI-VII	−2	−76	−31					> 20						> 20	
Class 3	(9) L AIns	−29	22	−2	L ant insula	−32	23	2	5.1	recog	L ant insula	−30	19	3	5.9	recog
	(10) R AIns	32	24	1	R ant insula	33	23	−1	2.4	recog	R ant insula	30	21	5	5.4	recog
	(16) L FO	−33	19	7	L ant insula	−32	23	2	6.5	recog	L ant insula	−30	19	3	5.0	recog
	(17) R FO	36	23	6	R ant insula	33	23	−1	7.6	recog	R ant insula	30	21	5	6.4	recog
	(26) R MCgG	6	18	37	R ant cingulate	6	23	35	5.4	recog	R ant cingulate G	9	20	39	4.1	accum
	(27) L MFG	−39	7	31	L post inf frontal G	−46	−2	35	12.1	accum	L inf frontal G	−43	0	35	9.0	accum
	(31) L MSFG	−4	27	42					> 20		L ant cingulate G	−5	24	44	3.7	recog
	(32) R MSFG	7	27	37	R ant cingulate	6	23	35	4.6	recog	R ant cingulate G	9	20	39	7.5	accum
	(36) L OpIFG	−41	11	25	L post inf frontal G	−46	−2	35	17.1	accum	L inf frontal G	−43	0	35	15.0	accum
	(37) R OpIFG	47	12	30	R inf frontal G	44	4	36	10.4	accum	R inf frontal G	39	4	31	11.4	accum
	(38) L OrIFG	−30	27	−1	L ant insula	−32	23	2	5.4	recog	L ant insula	−30	19	3	8.9	recog
	(39) R OrIFG	34	28	−1	R ant insula	33	23	−1	5.1	recog	R ant insula	30	21	5	10.0	recog
	(40) L Pallidum	−17	0	1	L striatum	−11	7	6	10.5	recog					> 20	
	(44) L PrG	−43	5	31	L post inf frontal G	−46	−2	35	8.6	accum	L inf frontal G	−43	0	35	6.4	accum
	(49) L SMC	−4	9	52	L med frontal G	−1	12	56	5.8	recog	L med frontal G	−7	11	53	3.7	recog
	(50) R SMC	7	14	50	R med frontal G	1	25	47	12.9	recog	R med frontal G	5	13	52	3.0	recog
	(58) L TrIFG	−39	30	14	L mid frontal G	−44	28	28	15.0	accum	L ant insula	−30	19	3	18.0	recog
	(61) R Ventral DC	20	−25	−6					> 20						> 20	
	(63) Cerebellar Verm. Lob. VIII-X	0	−55	−37					> 20						> 20	
Class 4	(12) L Caudate	−14	−1	15	L striatum	−11	7	6	12.4	recog					> 20	
	(13) R Caudate	16	0	16	R striatum	12	6	4	14.0	recog					> 20	
	(41) R Pallidum	18	5	0	R striatum	12	6	4	7.3	recog					> 20	
	(46) L Putamen	−22	3	−2	L striatum	−11	7	6	14.2	recog	L ant insula	−30	19	3	18.6	recog
	(47) R Putamen	23	7	−1	R striatum	12	6	4	12.1	recog	R ant insula	30	21	5	16.8	recog

Coordinates of all the decision-related regions described in the two papers by Ploran et al. (2007, 2011) were extracted, and the nearest region to each region of the present study within a limited distance (dist.) of 20 mm was determined. See Table 2 for the regions of the present study. As for the regions of Ploran and colleagues, the Talairach coordinates were transformed into the MNI coordinates for the distance calculation. No near regions within 20 mm (>20) are left blank. The region names followed the original Ploran papers, with the abbreviations: L, left; R, right; G, gyrus; S, sulcus; ant, anterior; post, posterior; inf, inferior; mid, middle; med, medial. The classes are described in the Introduction: sensory processor (sens), accumulator (accum), and moment-of-recognition region (recog). While Ploran et al. (2011) updated the concept of recog class to imply the commitment to a recognition decision by renaming to “comm,” we use “recog” here for simplicity of comparison. Ploran et al. (2011) introduced the fourth class of inferior temporal (IT) region although stimulus selectivity of the regions was not identified. The dominant class of the Ploran papers corresponding to each class of the present study was: sens to Class 1 (63% of the Class 1-nearst regions of the Ploran papers were sens), accum to Class 2 (68%), recog to Class 3 (66%), and recog to Class 4 (100%).

**Figure 10.**
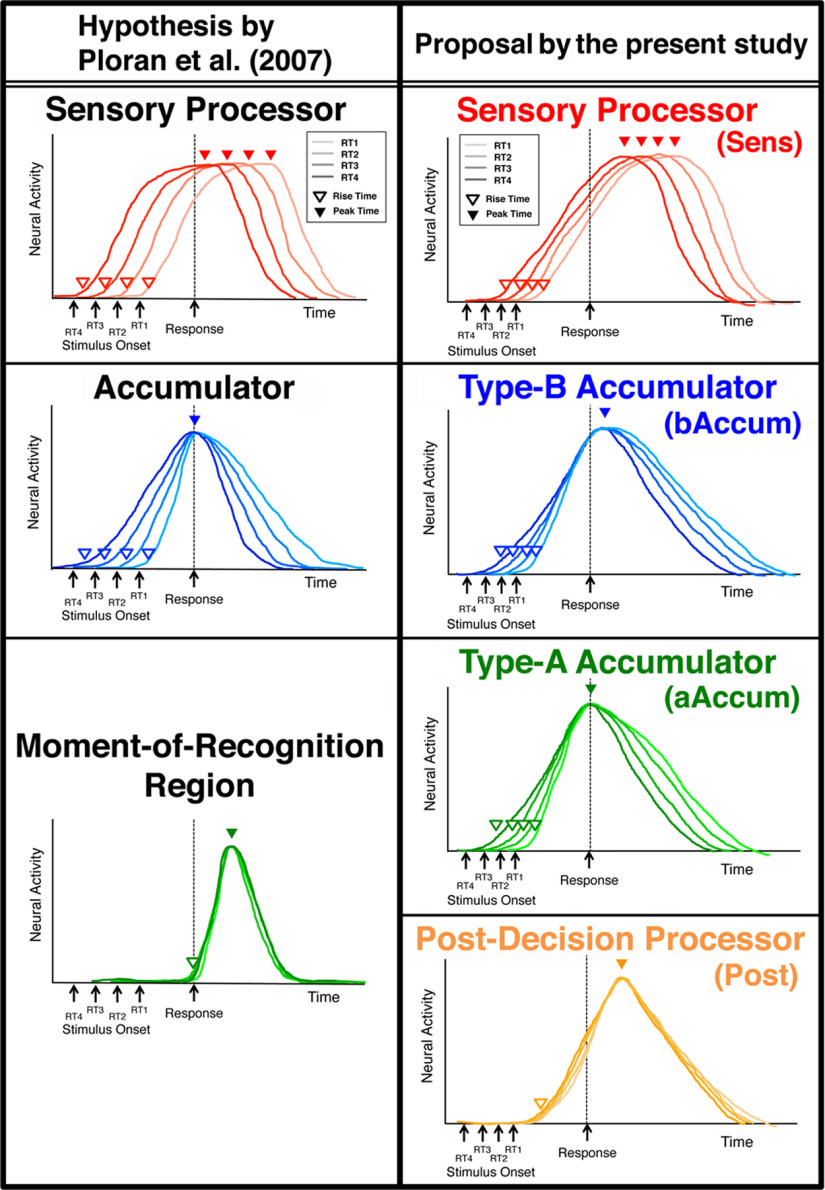
Proposal for the region classification by the present study in relation to the hypothesis by Ploran et al. (2007). The hypothesis by Ploran et al. (2007), whose original is drawn as BOLD signals on the stimulus-locked coordinates in Figure 2 of that paper, was redrawn as neural activities on the response-locked coordinates. The correspondence of the present proposal to the Ploran hypothesis was based on the anatomic correspondence shown in Table 4. Properties of the classes are described in the main text.

Despite the above correspondence, the present results also showed some discrepancies with the hypothesis by Ploran et al. (2007). In this hypothesis, to which we would refer below as the Ploran hypothesis, the moment-of-recognition regions are recruited at the moment of recognition so that their activity would peak after the response. If Class 3 and Class 4 share these properties of the moment-of-recognition region, both of the two classes are characterized by activity rises near the response moment and by activity peaks after the response. In the present results, however, Figure 9*C* shows significant differences between Class 3 and Class 4 in Peak_rsp_MN (the fourth panel) and in Rise_stm_MN (mean of the stimulus-locked rise times over RT groups; the fifth panel). Whereas Class 4 regions seemed to share the properties of the moment-of-recognition region, Class 3 regions showed the stimulus onset-dependent rise times similarly to Classes 1 and 2 (the fifth panel) and the peak times matching the response moment (the fourth panel). In addition, Peak_rsp_MN of Class 3 was even earlier than that of Class 2 whose correspondence is accumulator of the Ploran hypothesis (in the fourth panel). Facing these results, we reconsidered the classification of regions. In the present results, both Class 2 and Class 3 could be regarded as accumulators because their activity rose at the stimulus onset and increased toward the decision moment. These classes were differentiated in the activity peak times at and behind the decision moment for Class 3 and Class 2, respectively. Thus, we would propose to refer to Class 3 as “type-A accumulator (aAccum)” and Class 2 as “type-B accumulator (bAccum),” after the activity peaking “at” and “behind” the decision moment, respectively. In Class 4, both rise and peak times were dependent on the decision moment, which matched the characteristics of the moment-of-recognition region of the Ploran hypothesis. Emphasizing that their peaks were markedly behind the decision moments in our results, however, we would like to refer to Class 4 as “postdecision processor (Post).” We refer to Class 1 as “sensory processor (Sens),” whose definition is exactly the same as the Ploran hypothesis. Figure 10 shows our proposal for the region classification in relation to the Ploran hypothesis, illustrating that the different classifications are based on the subtle differences in the peak and rise times. The deconvolved signals seem to provide improved availability of temporal information such as whether the signal peaks are at the decision moment or behind, which could be implicit in BOLD signals. In Figure 10, the aAccum and bAccum regions do not differ in Rise_stm_MN, providing a reason to employ Slope_rsp_MN instead of Rise_stm_MN to separate these two classes, as described above. In a logical sense, however, Slope_rsp_MN and Rise_stm_MN can have equal power to cluster these regions because different Slope_rsp_MN could be derived from the same Rise_stm_MN and the different Peak_rsp_MN, the latter of which was employed as another clustering parameter. On the contrary, the results demonstrated that clustering is more effective when employing two different parameters (Peak_rsp_MN and Slope_rsp_MN) than employing one different (Peak_rsp_MN) and one same (Rise_stm_MN) parameter. We think that the results showing “two is better than one” justify the heuristic method to select the classification parameters in the present study. It should be noted that not all of the accumulators found in the results seemed to determine the decision timing because activity peaks of the bAccum regions were significantly behind the decision responses. Since activity peaks of the aAccum regions consistently matched the decision moments, it seems reasonable to postulate that decisions are made within the aAccum regions. In Discussion, we discuss this crucial issue regarding the relationship between accumulation and decision making.

### Activity profiles of CSRs in reference to classification of NCSRs

**Figure 11.**
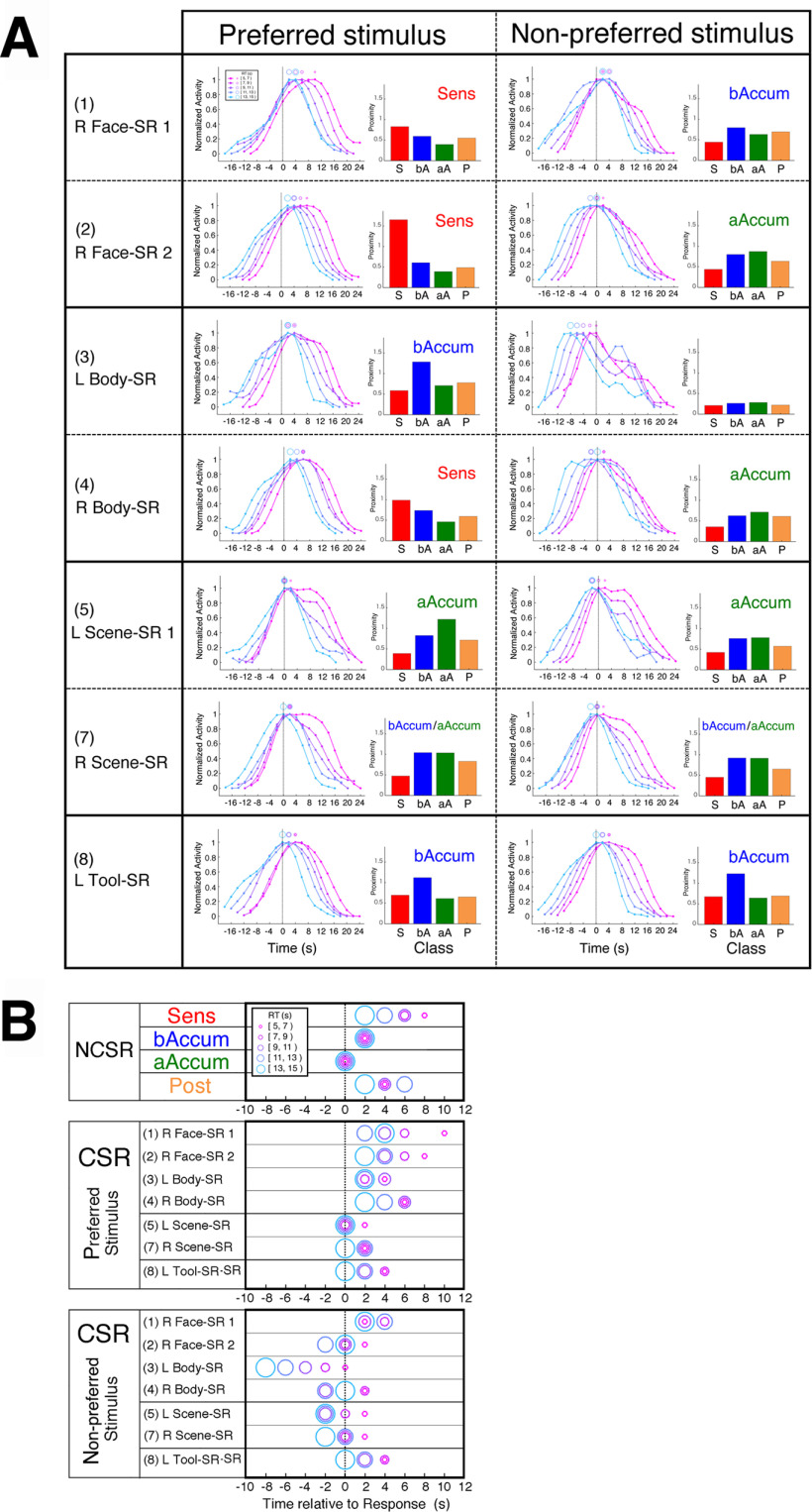
Activity profiles of CSRs. ***A***, Time courses of CSR activities averaged separately for the regions' preferred and nonpreferred stimuli on the response-locked coordinates. (See Table 1 for the list of the regions.) The activity profiles were classified into the four classes obtained for the NCSRs using the proximity to the classes defined in the same way as Figure 9*A*. In the estimation of the proximity in the four-dimensional clustering space, the clustering parameters such as Peak_stm_SD were scaled using the same scaling ratios as those of NCSRs in Figure 8*C*. The class was given by the largest proximity as indicated in each panel, whose axis is labeled with abbreviations: S (sensory processor), aA (type-A accumulator), bA (type-B accumulator), and P (postdecision processor). Classes whose difference in the proximity was <0.01 were both described, e.g., bAccum/aAccum. L Body-SR showed outstandingly small proximity to any of the classes for nonpreferred stimuli, so that this case was not classified. ***B***, Activity peak times on the response-locked coordinates, Peak_rsp, for each of RTs. The values were extracted from the class-averages of NCSRs (Fig. 9*A*) and from each CSR separately with preferred and nonpreferred stimuli (panel ***A***).

Figure 11*A* shows the time courses of CSR activities, which were averaged separately across trials with preferred and nonpreferred stimuli in each region. The CSRs were activated even for their nonpreferred stimuli as also observed by Tremel and Wheeler (2015) and Dunovan and Wheeler (2018), suggesting that they are not passively activated by visual stimuli but actively commit to the decision process. We characterized the activity profiles of CSRs in reference to the classification of NCSRs by estimating their proximity to each of the four NCSR classes [Sens (sensory processor), aAccum (type-A accumulator), bAccum (type-B accumulator), and Post (postdecision processor)] in the same way as in Figure 9*A*. Figure 11*A* reveals that the classes of CSRs depended not only on region but also on stimulus preference. The four regions of Face-SRs (face-selective regions) and Body-SRs (body-selective regions) showed quite different activity profiles between the preferred stimulus (human image) and nonpreferred stimuli (scene and tool images). In particular, L Body-SR was classified as bAccum for the preferred stimulus, whereas for the nonpreferred stimuli it was not close to any of the classes showing very small proximity values. Curiously, the activity peaks of L Body-SR for the nonpreferred stimuli were outstandingly earlier than the response moment. The remaining three of the Face-SRs and Body-SRs were classified as aAccum or bAccum for the nonpreferred stimuli while they were classified as Sens for the preferred stimuli, indicating their continued activity for the preferred stimuli after the decision. On the other hand, the three regions of Scene-SRs (scene-selective regions) and Tool-SR (tool-selective region) showed less different activity profiles between preferred and nonpreferred stimuli, classified as aAccum or bAccum regardless of stimulus.

Although the results showed the variety of activity patterns of the CSRs, we would propose that the fundamental classes of CSRs are accumulators because their rise and peak times were stimulus and response dependent, respectively, and they were classified as accumulators (aAccum and bAccum) in most cases. The property characteristic to the CSRs as accumulators is that their activities are modified by their selectivity to stimuli. It is noteworthy that their peaks were earlier for nonpreferred stimuli than for preferred stimuli in most cases and often earlier than even the response (decision) moment as shown in Figure 11*B*, which was rarely observed in the NCSRs. Such distinguishing properties of the CSRs plausibly reflect their commitment to competing processes among the alternatives of decision. It seems reasonable to regard their peaks before the decision moment for nonpreferred stimuli as their withdrawals from the losing competition. In this sense, L Body-SR might show peculiarly early withdrawals from the losing competition. In contrast to the cases for nonpreferred stimuli, the activity peaks of all the CSRs for the preferred stimuli were at the RT or later, which could be reasonably interpreted as activities of the competition winners that do not drop before the decision. The different classifications as Sens (the Face-SRs and Body-SRs) and aAccum/bAccum (the Scene-SRs and Tool-SRs) for preferred stimuli might originate from difference in intrinsic properties of the objects for which the regions were selective. As a possible explanation, recognition of humans would recruit further processing of the image contents such as facial expression and social categorization by age and sex, while recognition of scene would make attention more divergent. In such a case, Face-SRs and Body-SRs for their preferred stimuli would keep activity after the decision for the extended processing, which could be classified as Sens. Based on the observed properties of CSRs, we would propose that their fundamental class is the content-specific accumulator as proposed by Tremel and Wheeler (2015). They seem to play a distinctive role in representing competition among the choice alternatives, so that their activity profiles could be modified depending on the dynamics of the competition, including withdrawals as the losers. In addition, the CSRs could reflect processing specific to their preferred objects, such as extended processing of human stimuli after the decision. Because these distinctive properties of CSRs cannot be covered by a single class of either type-A or type-B accumulator, we would refer to the class of the decision-related CSRs as “type-C accumulator (cAccum)” after “content-specific accumulator.”

## Discussion

### Classification of decision-related regions and proposal for a concept of accumulation system

Previous decision-making studies have found neural substrates of evidence accumulation in multiple brain regions of animal and human, raising questions about their functional relations in decision processes. To work on this issue, we employed the fMRI deconvolution to take advantage of the whole-brain coverage with improving availability of the temporal information of activity, which is critical to identify the accumulators. In our results of the visual category-decision experiment, the NCSRs were classified into the four groups of Sens (sensory processor), aAccum (type-A accumulator), bAccum (type-B accumulator), and Post (postdecision processor) regions. From their activity profiles (Fig. 10), the Sens regions and Post regions were associated in the antecedent and subsequent processing to the accumulation, respectively. Although both aAccum and bAccum regions showed the accumulation profiles, only aAccum regions showed activity peaks consistently matched the decision moments, reasonably indicating that decisions are made within aAccum regions. Regarding the decision-related CSRs, whose class was given as cAccum (type-C accumulator), their activity profiles were modified by their selectivity to stimuli, suggesting their commitment to competition among the alternatives in decision making.

**Figure 12.**
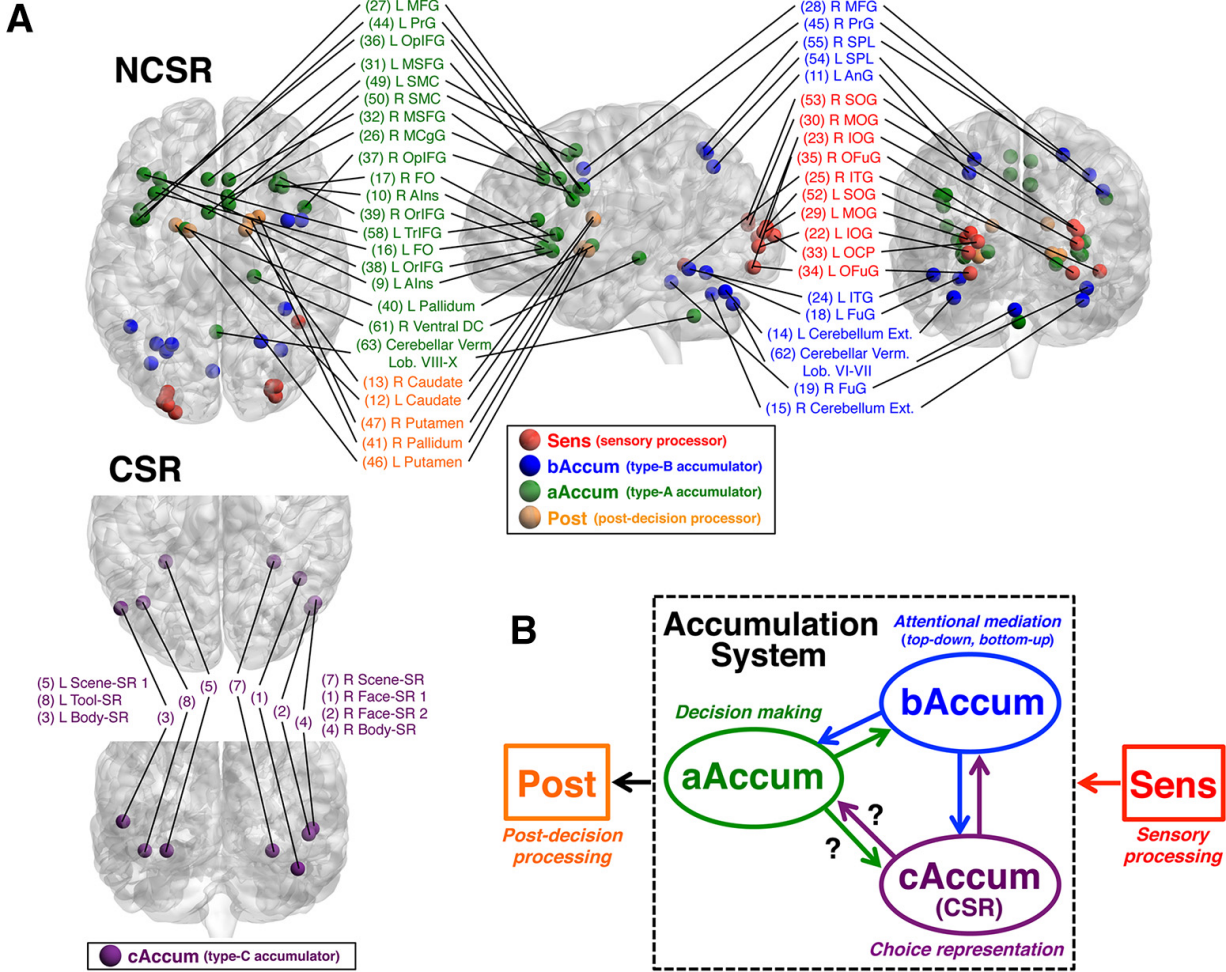
Organization of the decision-related regions over the brain. ***A***, Anatomical distribution of the classes of NCSRs and CSRs. The region indices and abbreviations are consistent with Tables 1 and 2. For CSRs, parts of the superior and posterior views are shown. ***B***, A schematic drawing of our proposal of the accumulation system. Sens and Post are regions associated with the antecedent and subsequent processing to accumulation, respectively. Evidence accumulation is conducted by the system comprising type-A, type-B, and type-C accumulators, which work at different levels of information abstraction in a co-evolving manner. The system concept may provide a clue to unravel the puzzle of the universal applicability of the accumulation account over a variety of decision making. (The rationale is described in the main text.)

Now, how do these classes of regions work in the decision process? Figure 12*A* shows the anatomic distribution of the classes of regions, which evidently illustrate a posterior-anterior gradient over the brain; Mostly Sens in the occipital areas, bAccum and cAccum in the temporal and parietal areas, aAccum in the frontal areas, and Post in the subcortical structures. This spatial gradient appears to imply hierarchical processing from sensory to abstract information along the organization from occipital to frontal areas. In general, hierarchical processing includes interactions between the different levels through feedback as well as feedforward connections (Felleman and Van Essen, 1991). Thus, in particular, the interactions among the three classes of accumulators may play an important role in the accumulation process. According to the knowledge of previous studies of the parietal cortex, its posterior regions are reciprocally connected with the prefrontal and temporal cortices including the high-level visual areas (Steinmetz, 1998). It is well known that the posterior parietal cortex plays an essential role in attentional functions, which mediate associations of perceptual analysis and actions in a wide variety of tasks. In other words, the parietal cortex selects particular components (e.g., motion, color, object) from multidomain sensory information in a manner appropriate to the task requirements represented in the frontal cortex (for review, see Gottlieb, 2007; Shomstein, 2012). In this context, bAccum regions in the parietal cortex may operate on cAccum and bAccum regions of the temporal cortex to extract the high-level visual information that is required by aAccum regions of the frontal cortex. Since activities of bAccum and cAccum regions depended on the decision moment and competition process, bAccum regions may receive control signals from aAccum regions and also send regulatory signals to cAccum regions. This idea of the interacting accumulators seems to be consistent with the present observation of the peak-time variations (Fig. 11*B*), which suggest that the accumulation process is not steadily passive but actively controlled on the way to decision.

Based on these grounds, we would propose an idea that the accumulation process is conducted by multiple accumulator regions of heterogeneous functions working in a distributed manner as a system, to which we would refer as the accumulation system. A possible organization of the accumulation system is schematically shown in Figure 12*B*. In this proposal, cAccum regions separately accumulate choice-specific information being controlled by bAccum regions, which extract the information components required by aAccum regions. Considering the integrative functions of the parietal cortex over a wide variety of stimuli and actions, bAccum regions convert information from cAccum regions into some abstract forms to send to aAccum regions. Then (a part of) aAccum regions make decisions based on comparisons among the choices in the abstract forms, and terminate their demand signals of accumulation to the other accumulators. Because the abstraction levels of information represented by the three classes of accumulators are different, the information accumulation needs to be conducted at each level in a co-evolving manner, which constitutes the core idea of this system concept.

### Universality of the accumulation account over a variety of tasks

The concept of the accumulation system may provide one step to solve a crucial puzzle of the wide applicability of the accumulation model over a variety of decision tasks. The accumulation model has been demonstrated to be effective for the tasks over modalities and contents of sensory stimuli; for example, visual tasks such as random-dot motion direction discrimination (Palmer et al., 2005), symbol density discrimination (Ratcliff et al., 1999), object distance discrimination (Ratcliff et al., 2003), brightness discrimination (Ratcliff et al., 2007), and object category discrimination (Philiastides et al., 2006; Murata et al., 2014), somatosensory tasks such as vibrotactile frequency discrimination (Mulder and van Maanen, 2013), and auditory tasks such as phonetic discrimination (Binder et al., 2004). In these tasks, regions representing the alternatives vary in a wide range; for example, the alternatives of visual object categorization are represented by the high-level visual areas, while the alternative phonemes of the phonetic discrimination are represented by the auditory cortex (Binder et al., 2004). Thus, the crucial question arises: How can such different kinds of perceptual decisions be neurally implemented in a manner unified by the accumulation model? As another important knowledge to consider this question, some regions have been found to be commonly associated with a wide range of decision tasks. For example, the anterior insula is reported to be engaged in motion-direction discrimination (Ho et al., 2009; Liu and Pleskac, 2011), bar-length categorization (Grinband et al., 2006), facial-expression discrimination (Thielscher and Pessoa, 2007), phonetic discrimination (Binder et al., 2004), object identification (Ploran et al., 2007, 2011), and object categorization (Tremel and Wheeler, 2015). If (a part of) aAccum regions work commonly to various decision-making tasks, their working properties can determine the properties common to such various decisions, or in other words, implementation of the accumulation model by aAccum regions can cause the universality of the model. In a similar context to this discussion, a concept of “general accumulator” (Heekeren et al., 2004; Ploran et al., 2011) has been proposed to indicate accumulators working commonly to various tasks, being differentiated from domain-dependent accumulators. The general accumulators were reported to be mostly in the frontal regions, such as the right insula (Ho et al., 2009), anterior insula and inferior frontal regions (Liu and Pleskac, 2011), and left inferior frontal sulcus (Filimon et al., 2013). It should be noted that their cortical locations are nearby those of aAccum regions of this study (Fig. 12*A*). Thus, it seems to be an idea worth exploring that the decision-making region (decision maker) corresponds to a general accumulator in the frontal cortex. This picture would be consistent with the report of Ho et al. (2009), which found neural correlates of modality-independent decision variables of the accumulation model in the insula of the frontal cortex. In an extended idea combined with our accumulation-system concept, the general accumulators receive a certain abstraction level of evidence information through the accumulation system, accumulate information according to the accumulation model, and make decisions in a universal manner regardless of sensory modality. Fundamental tests of our proposal of the system would be to determine the degrees of simultaneity of decision moments and aAccum activity peaks over a variety of decision tasks. Despite of our suggestion about the decision makers in the aAccum class, we neither argue that all aAccum regions are decision makers nor deny the possibility that a single or very restricted regions of the class are decision makers. Although a certain aAccum region that generates activity peaks most closely to decision moments is most likely to be the decision maker, the precision of the present study was not high enough to determine differences within the aAccum class.

The accumulation-system concept could be a viewpoint to address the recent emerging issues of involvement of the parietal regions in decision making (Pisupati et al., 2016). Studies of rat and monkey showed that the inactivation of the decision-related prefrontal regions substantially impaired decisions while that of the posterior parietal regions did little or not, suggesting that the parietal cortex, despite its accumulating activity, does not causally contribute to decision making but plays a supporting role (Erlich et al., 2015; Hanks et al., 2015; Katz et al., 2016). Another rat experiments showed that inactivation of the posterior parietal cortex impaired visual decisions but not auditory decisions, suggesting that the involvement of parietal regions depends on the task modality (Raposo et al., 2014; Licata et al., 2017). From the system viewpoint, it is possible that the parietal regions contribute to the evidence accumulation with the task-relevant weights but do not make decisions themselves. In this sense, accumulation activity would be a necessary but not sufficient sign of a decision-making region.
